# Impact of Field-of-view Zooming and Segmentation Batches on Radiomics Features Reproducibility and Machine Learning Performance in Thyroid Scintigraphy

**DOI:** 10.1097/RLU.0000000000005995

**Published:** 2025-06-17

**Authors:** Soroush Bagheri, Ghasem Hajianfar, Maziar Sabouri, Omid Gharibi, Babak Yazdani, Atena Aghaee, Ali Mohammad Nickfarjam, Akram Yazdani, Akbar Aliasgharzadeh, Habiballah Moradi, Arman Rahmim, Habib Zaidi

**Affiliations:** * Department of Medical Physics and Radiology, Allied Medical Sciences Faculty, Kashan University of Medical Sciences, Kashan, Iran; † Division of Nuclear Medicine and Molecular Imaging, Geneva University Hospital, Geneva, Switzerland; ‡ Department of Physics and Astronomy, University of British Columbia, Vancouver, BC, Canada; § Department of Integrative Oncology, BC Cancer Research Institute, Vancouver, BC, Canada; ‖ Department of Nuclear Medicine, Namazi Hospital, School of Medicine, Shiraz University of Medical Sciences, Shiraz; ¶ Department of Nuclear Medicine, Research Center, Mashhad University of Medical Sciences, Mashhad; # Health Information Management Research Center, Kashan University of Medical Sciences, Kashan, Iran; ** Department of Health Information Management and Technology, School of Allied Medical Sciences, Kashan University of Medical Science, Kashan, Iran; †† Autoimmune Diseases Research Center, Kashan University of Medical Sciences, Kashan, Iran; ‡‡ Department of Biostatistics & Epidemiology, Faculty of Health, Kashan University of Medical Sciences, Kashan, Iran; §§ Department of Nuclear Medicine and Molecular Imaging, University of Groningen, University Medical Center Groningen, Groningen, The Netherlands; ‖‖ Department of Nuclear Medicine, University of Southern Denmark, Odense, Denmark; ¶¶ University Research and Innovation Center, Óbuda University, Budapest, Hungary

**Keywords:** robust features, machine learning, nuclear medicine, radiomics, reproducibility, scintigraphy, thyroid

## Abstract

**Background:**

Thyroid diseases are the second most common hormonal disorders, necessitating accurate diagnostics. Advances in artificial intelligence and radiomics have enhanced diagnostic precision by analyzing quantitative imaging features. However, reproducibility challenges arising from factors such as the field-of-view (FOV) zooming and segmentation variability limit the clinical application of radiomic-based models.

**Aim:**

This study focuses on evaluating the impact of segmentation and FOV zooming on the reproducibility of radiomic features and improved performance of machine learning (ML) when using reproducible features for classification of thyroid scintigraphy images into normal, diffuse goiter (DG), multinodular goiter (MNG), and thyroiditis.

**Patients and Methods:**

A retrospective analysis was conducted on 872 thyroid scintigraphy cases from 3 centers. Radiomic feature reproducibility was assessed using the intraclass correlation coefficient (ICC), with robust features (ICC≥0.80) identified under segmentation and zooming conditions. Four ML training scenarios were implemented to train models on Center A data, including (1) all, (2) zoom-robust, (3) segmentation-robust, and (4) mutually robust features, with 3 feature selection methods and 7 classifiers. Models were validated on external data sets (centers B and C).

**Results:**

FOV zooming significantly reduced feature reproducibility (ICC≥0.80: 49%), while segmentation effects were minimal (ICC≥0.80: 96%). Models trained on mutually robust features outperformed those trained using all features. Boruta-MLP achieved the highest accuracy (0.71, *P*-value <0.001 vs. all features) in zoomed data sets, and RFE-MLP performed best (0.69, *P*-value <0.001 vs. all features) in the baseline data set, with Gray-Level Co-occurrence Matrix (GLCM) features frequently selected.

**Conclusions:**

Utilizing robust radiomic features significantly improved the performance of ML models in thyroid disease classification, enabling more accurate and generalizable diagnostic outcomes across diverse data sets.

Thyroid disease, the second most prevalent hormonal disorder, is experiencing a rising incidence while its mortality rate remains constant.^1^ Prevalent diagnostic techniques encompass sampling (eg, blood analyses, fine-needle aspiration), and imaging using ultrasound (US), computed tomography (CT), magnetic resonance imaging (MRI), and single-photon emission computed tomography (SPECT) modalities.^2–4^ US imaging is primarily employed for the evaluation of thyroid nodules. Nonetheless, its subjective nature and reliance on the operator have led to restricted interobserver variability. Although there is good concordance in inter-reader ultrasound image interpretation, interobserver variability remains challenging even among less experienced observers.^5^ CT and MRI assess structural abnormalities but frequently produce nonspecific findings, particularly in conditions such as Graves disease, and hence are rarely used.^6^ Thyroid scintigraphy using ^99m^Tc-pertechnetate and ^123^I serves a crucial role in the assessment of the thyroid gland status. While ^99m^Tc-pertechnetate is frequently utilized owing to its accessibility and cost-effectiveness, ^123^I is commonly favored in clinical practice for thyroid scintigraphy, especially in specific regions and diagnostic protocols.^7–18^

The necessity of accurate diagnosis in the shortest time possible is of great importance in medicine. Hence, artificial intelligence (AI) and radiomics have been widely adopted to accelerate medical decision processes.^19,20^ The field of radiomics involves the extraction and analysis of quantitative features from medical images through sophisticated algorithms to reveal patterns that can assist in diagnosis, prognosis, outcome prediction, and treatment planning.^21,22^ Another reason compelling us to adopt AI technologies is that noninvasive techniques, such as US and NM, exhibit limited sensitivity and specificity.^23–25^ Notwithstanding the burgeoning promise of radiomics, obstacles persist, particularly with the evaluation of repeatability and reproducibility.^26–28^ According to research and guidelines for identifying biomarkers, it is crucial to assess the repeatability, reproducibility, and prevalent robust characteristics of these indicators before making clinical judgments. A radiomic feature must be robust under batch effects, exhibiting consistency between 2 measurements under varying settings to qualify as a reliable clinical biomarker. Popular batches in medical imaging and radiomics workflow include changes in equipment, data acquisition, software, segmentation, operator, and sample.^29^

In thyroid scintigraphy imaging, certain issues, such as differential diagnosis between a normal thyroid and a diffuse goiter (DG), are challenging and may occasionally be overlooked.^15,30–32^ To mitigate this issue, images are often captured at higher magnification. Even in some centers where the devices are not capable of higher zoom imaging, pinhole collimators are used, primarily for the better identification of different thyroid diseases, such as multinodular goiter (MNG), thyroiditis, etc.^13,14,33,34^ Changes in zooming or field-of-view (FOV) can introduce batch effects on radiomic features.^31,35,36^

This study aimed to evaluate the batch effects of segmentation and FOV zooming on radiomic features’ reproducibility and machine performance in image classification using thyroid scintigraphy images. To this end, we will identify reliable features in the first step, and then classify the images into 4 categories—normal, DG, MNG, and thyroiditis, once using all features, and in a separate attempt, using only the reliable ones.

## PATIENTS AND METHODS

**FIGURE 1:**
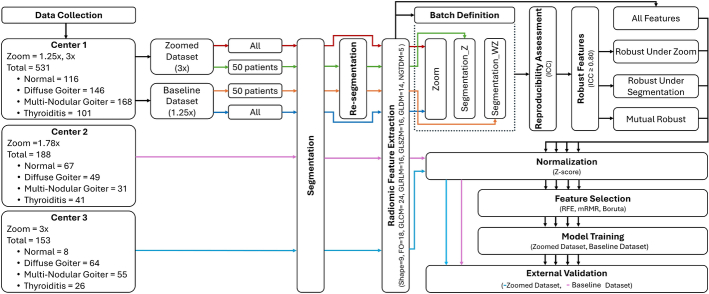
Detailed steps of the study protocol at one glance.

The flowchart of the study is shown in Figure 1. In what follows, we elaborate on the various steps.

### Data Collection

This retrospective study analyzed 872 cases collected from 3 centers between 2021 and 2023 (center A: 531 cases, center B: 188 cases, center C: 153 cases). Of the total cases, 62% were women: center A (75%), center B (27%), and center C (67%). Each case in center A included 2 images captured at different zoom levels: one at ×1.25 and the other at ×3. However, images of patients from center B and center C were obtained only at ×1.78 and ×3, respectively. We mention images with ×1.25 and ×3 magnifications as baseline and zoomed data sets, respectively. Patients with single-zoom imaging, low counts, or lead shields in their images were excluded from the study. The research was conducted with ethics approval (code: IR.KAUMS.NUHEPM.REC.1403.022).

### Image Acquisition

Anterior thyroid scintigraphy images were acquired using a gamma camera with a matrix size of 128×128 pixels, ~15–20 minutes after patients received an injection of 6–10 mCi of the ^99m^TC-pertechnetate radiopharmaceutical. Patients were classified into 4 categories based on clinical reports: MNG, thyroiditis, DG, and Normal. The scans showing diminished or negligible uptake in the presence of suppressed TSH were classified as indicative of subacute thyroiditis. However, other less common differential diagnoses, such as iodine-induced hyperthyroidism, thyrotoxicosis factitia, and struma ovari, were also considered.^34,37^

### Radiomic Feature Reproducibility Assessment

To assess the reproducibility of radiomic features, we only used the data from center A and defined 2 batches: segmentation and zoom. The segmentation batch aims to investigate the impact of intraobserver segmentation variability on radiomic features’ reproducibility, while the zoom batch explores the effect of FOV magnification. Then, the 2-way mixed average measure or (3, k) intraclass correlation coefficient (ICC)^38^ score was calculated for each feature within each of the batch groups assigning them to a group of excellent (0.90≤ICC≤1.00), good (0.80≤ICC<0.90), fair (0.50≤ICC<0.80), and poor (ICC<0.50) reproducibility based on their ICC scores.

The zoom batch includes all the available samples (531 patients) at ×1.25 and ×3 zooms. However, the segmentation batch incorporates 50 samples (50% normal) segmented twice by the same physician with 4 years of experience, with a 2-month gap between the 2 attempts. All abnormal categories were combined into a single group in this batch. Also, segmentation was performed separately for images at ×1.25 and ×3 zoom levels, giving segmentation without zoom (Segmentation_WZ) and segmentation with zoom (Segmentation_Z) subgroups.

In this study, segmentation and radiomic feature extraction were performed using 3D-Slicer software and the Pyradiomics library.^39^ A list of all 102 radiomic features extracted is provided in the Supplementary Section, Table S1, Supplemental Digital Content 1, http://links.lww.com/CNM/A565.

### Feature Selection and Classification

To explore the impact of feature reproducibility on classification performance, we grouped radiomic features into 4 categories: all features, features reproducible under zoom changes, features reproducible under segmentation variations, and mutually robust features, which were mutually reproducible under both segmentation and zoom batches.

The training process was separately performed on baseline and zoomed images of patients from center A in 4 scenarios corresponding to the feature set used for training. Scenario 1 included all extracted features, scenario 2 involved features reproducible under zoom changes, scenario 3 contained features reproducible under segmentation variations, and scenario 4 comprised mutually robust features. In each scenario, the features were first normalized with z-score normalization and then subjected to feature selection using 3 methods: boruta, recursive feature elimination (RFE) with random forest (RF) core, and minimum redundancy maximum relevance (MRMR).

The selected features were then processed through internal 5-fold cross-validation, during which 7 classifiers were used. Decision tree (DT), k-nearest neighbors (KNN), multilayer perceptron (MLP), naive bayes (NB), RF, support vector machine (SVM), and extreme gradient boosting (XGB) were applied alongside hyperparameter optimization. The hyperparameters were optimized with grid search and 5-fold cross-validation.

After training, the models were tested on 2 external data sets from centers B and C. The data from center B, containing thyroid images at ×1.78 zoom, was used as the external validation for the training set with baseline zoom, while the data from center C, with ×3 zoom, validated the zoomed training set. Before external validation, the features extracted from the 2 external sets were normalized using the mean and SD of features from the corresponding training sets. Then, each model was separately applied to the external data set.

### Model Evaluation

Model evaluation was conducted using various performance metrics, including accuracy, area under the receiver operating characteristic curve (AUC-ROC), precision, recall, and F1 score. These metrics were calculated under 3 different averaging methods: macro, which treats all classes equally; micro, which considers the global performance across all instances; and weighted, which accounts for class imbalance by assigning weights proportional to class frequencies. Wilcoxon rank sum test with 1000 bootstraps on accuracy was used to find significantly different (*P*-value<0.05) between all feature-trained models and mutual rubost features.

## RESULTS

### Data Collection

TABLE 1 summarizes information about patients' demographics from various centers, including age, gender, the number of distinct classes they belong to, and the vendor.

**TABLE 1:** Patient Information Categorized by Center

			Age	Gender			Disease			Total	Vendor Name
Data Set	Center	Zooming	Mean±SD	Female	Male	Diffuse Goiter	Multinodular Goiter	Normal	Thyroiditis	N	Manufacture
Train	A	×1.25×3.0	43±13	394	137	146	168	116	101	531	MEDISO
External	B	×1.78	NA	51	137	49	31	67	41	188	GE INFINIA
External	C	×3.0	47±5	103	50	64	55	8	26	153	SIEMENS
Total				548	324	259	254	191	168	872	

### Image Acquisition

**FIGURE 2:**
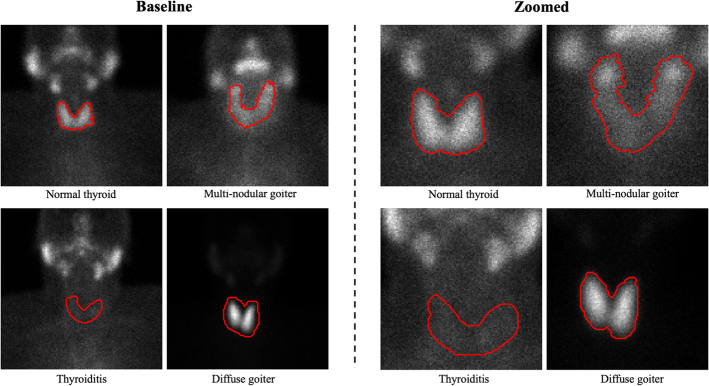
Baseline and zoomed image samples along with their segmentations. Zoomed and baseline samples of each class correspond to the same patient.

Figure 2 illustrates segmented examples of the 4 classes. In each class, the baseline and zoomed images correspond to a single patient.

### Radiomic Features Reproducibility Assessment

**FIGURE 3:**
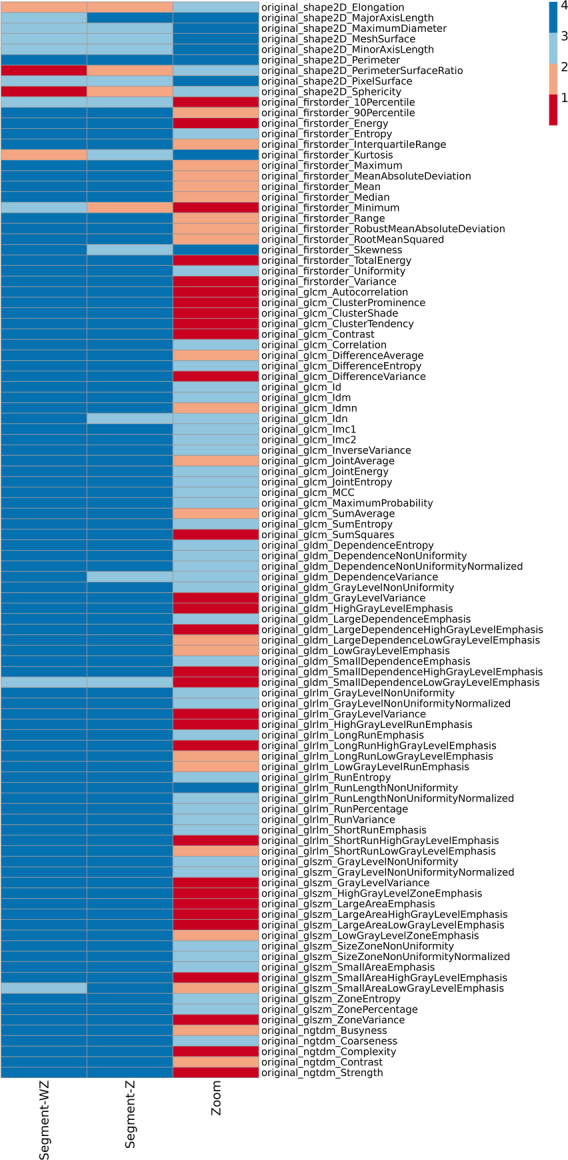
Behavior of individual features across segmentation (with and without zoom) and the zoom batches (1: ICC<0.50, 2: 0.50≤ICC<0.80, 3: 0.80≤ICC<0.90, 4: 0.90≤ICC≤1.00).

**FIGURE 4:**
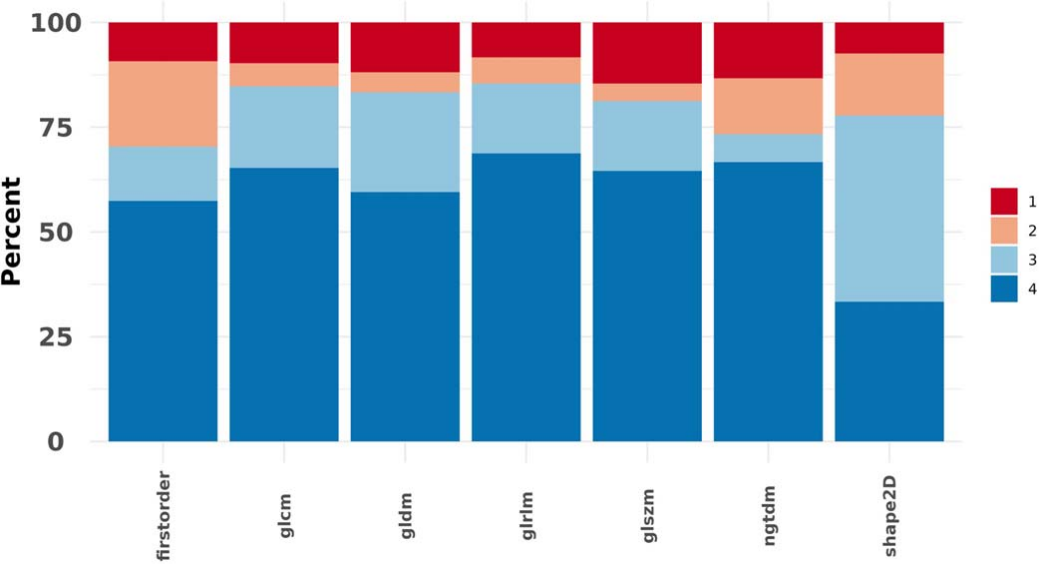
Overall variation of feature families across all batches (1: ICC<0.50, 2: 0.50≤ICC<0.80, 3: 0.80≤ICC<0.90, 4: 0.90≤ICC≤1.00).

**FIGURE 5:**
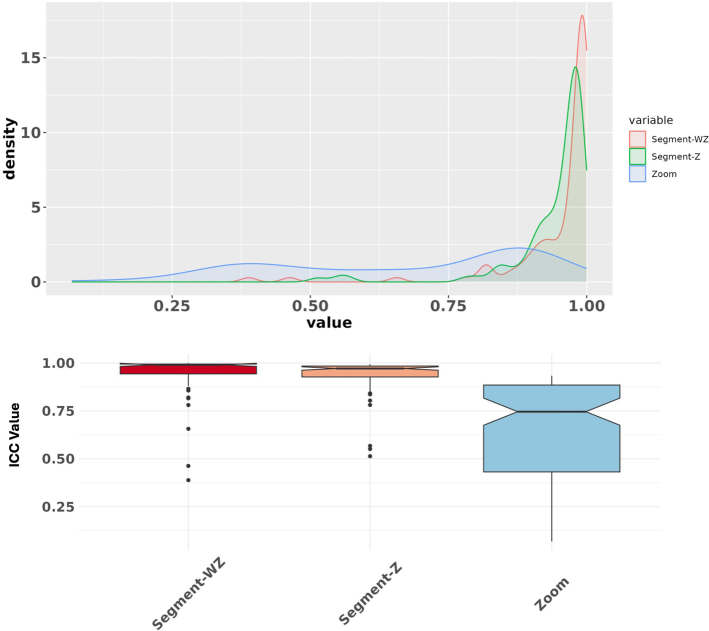
ICC score distribution across segmentation (with and without zoom) and zoom batches. Segmentation minimally interrupts feature reproducibility, while zoom leads to a more variable distribution.

Figures 3 and 4 present the results of the ICC analysis for individual features and different radiomic families under all batches. Overall ICC score variability across the 3 different batches is also shown in Figure 5. According to Figure 3, most of the radiomic features (96%) in both baseline and zoomed data sets remained reproducible (ICC≥0.80) under segmentation batch. However, this number dropped to only 49% under the Zoom batch. In addition, as shown in Figure 4, the behavior of features within the same family across all batches was largely consistent, with shape2D features exhibiting the highest variations.

In Figure 5, the density plots of both with and without zoom data show that the segmentation batch does not interrupt feature reproducibility significantly. This is evident by the sharp peak around ICC=1. Comparing segment-Z and segment-WZ shows that the impact of the segmentation batch is less pronounced in zoomed images, giving a more peaked distribution. In contrast, the zoom batch effect alone results in a smoother and more dispersed ICC score distribution. A smoother density under the zoom batch indicates that the ICC values are more distributed across a wider range, showing less agreement. This suggests that the zoom effect introduces variability in the feature values. The boxplot further supports this observation, as both segment-WZ and segment-Z lead to ICC scores tightly clustered near 1, with minimal spread and a few outliers. Meanwhile, zoom exhibits a broader spread and lower median, indicating higher variability.

### Feature Selection and Classification

TABLE 2 presents the mutually robust features with ICC≥0.80 across all batches. The features reproducible under each batch, along with all radiomic features used in this study, are presented separately in Supplementary Tables S2–S4, Supplemental Digital Content 1, http://links.lww.com/CNM/A565.

**TABLE 2:** Mutually Robust Features Across All the Batches in this Study

	Feature Family						
	Shape	First Order	GLCM	GLDM	GLRLM	GLSZM	NGTDM
Feature abbreviation	MaximumDiameter MeshSurface PixelSurface MinorAxisLength MajorAxisLength Perimeter	Entropy Uniformity Skewness	IMC1 ID IDM IMC2 MCC IDN DE Correlation JEnergy MP JEntropy SEntropy IV	DE GLNU LDE DV DNU DNUN SDE	GLNUN RLNUN GLNU RV LRE RP SRE RE RLNU	GLNUN SZNU GLNU ZE ZP SAE SZNUN	Coarseness

GLCM indicates gray-level co-occurrence matrix; GLDM, gray-level dependence matrix; GLRLM, gray-level run length matrix; GLSZM, gray-level size zone matrix; NGTDM, neighborhood gray-tone difference matrix.

**FIGURE 6:**
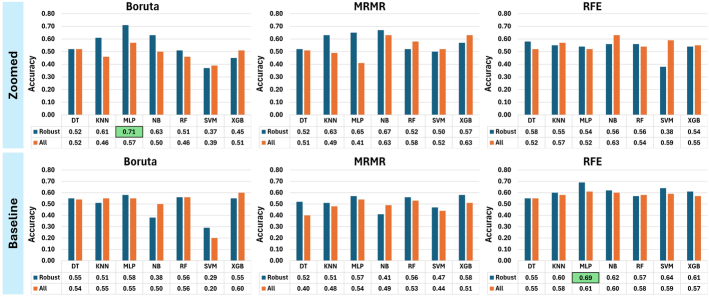
Comparison of the accuracy between all trained models in this study. “Zoomed” and “Baseline” refer to the zooming condition of the training and validation sets, while “Robust” and “All” mention whether mutually robust versus all features were used to train models. DT indicates decision tree; KNN, k-nearest neighbors; MLP, multilayer perceptron; MRMR, minimum redundancy maximum relevance; NB, naïve bayes; RF, random forest; RFE, recursive feature elimination; SVM, support vector machine; XGB, extreme gradient boosting.

The results for all scenarios are provided in Supplementary Tables S5–S20, Supplemental Digital Content 1, http://links.lww.com/CNM/A565, while the results for scenarios 1 and 4 are discussed here. This is because the primary objective of this study is to identify and highlight the impact of the most reliable features on classification performance, making the findings of scenario 4 particularly significant. We determined mutually robust features across 3 analyses: segmentation with zoom, segmentation without zoom, and zoom itself as a batch factor. Next, we trained machine learning (ML) models separately once using these mutually robust features and once using all the features extracted from both baseline and zoomed data sets. Finally, the trained models were evaluated on the external dataset with the correlated zooming setting as the training sets. Figure 6 summarizes the performance of each trained model in terms of accuracy.

**FIGURE 7:**
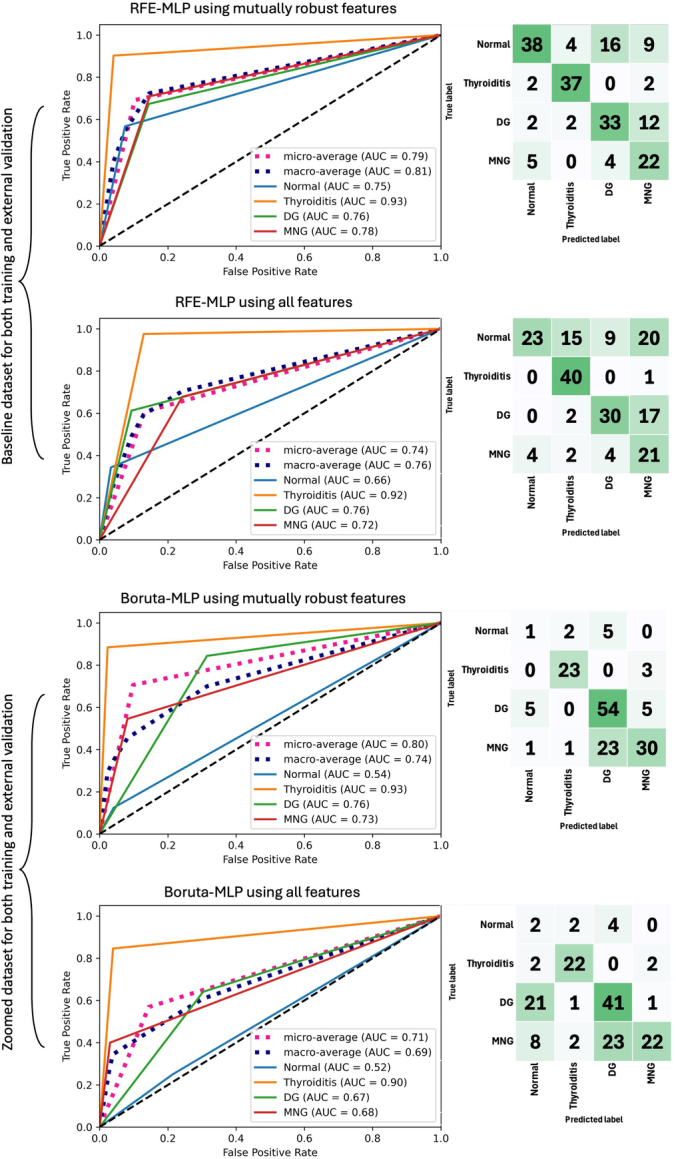
ROC curves and confusion matrices of the 2 top models trained and evaluated once with mutually robust features and once using all features on zoomed and baseline data sets.

When using zoomed data set for training and validation, Boruta-MLP trained with mutually robust features achieved the best result, yielding an accuracy of 0.71. On the other hand, when training and validation were performed on the baseline data set, RFE-MLP surpassed other models, achieving an accuracy of 0.69. The accuracy of the same models dropped to 0.57 and 0.61 for Boruta-MLP and RFE-MLP while using all features with *P*-value <2.2e-16, demonstrating a significant level of inferior machine performance. Detailed results of the confusion matrices and ROC curves of these 2 models are shown in TABLE 3 and Figure 7.

**TABLE 3:** Performances of the top models when trained with mutually robust features on baseline data (top) and zoomed data (bottom), and evaluated on the corresponding external datasets

Model	Zoom	Set	Class	Precision	Recall	F1-score	Support
RFE-MLP	Baseline dataset	Robust Features	Normal	0.81	0.57	0.67	67
			Thyroiditis	0.86	0.90	0.88	41
			DG	0.62	0.67	0.65	49
			MNG	0.49	0.71	0.58	31
			Micro Avg (Accuracy)	0.69	0.69	0.69	188
			Macro Avg	0.70	0.71	0.69	188
			Weighted Avg	0.72	0.69	0.69	188
		All Features	Normal	0.85	0.34	0.49	67
			Thyroiditis	0.68	0.98	0.80	41
			DG	0.70	0.61	0.65	49
			MNG	0.36	0.68	0.47	31
			Micro Avg (Accuracy)	0.61	0.61	0.61	188
			Macro Avg	0.65	0.65	0.60	188
			Weighted Avg	0.69	0.61	0.60	188
Boruta-MLP	Zoomed dataset	Robust Features	Normal	0.14	0.12	0.13	8
			Thyroiditis	0.88	0.88	0.88	26
			DG	0.66	0.84	0.74	64
			MNG	0.79	0.55	0.65	55
			Micro Avg (Accuracy)	0.71	0.71	0.71	153
			Macro Avg	0.62	0.60	0.60	153
			Weighted Avg	0.72	0.71	0.70	153
		All Features	Normal	0.06	0.25	0.10	8
			Thyroiditis	0.81	0.85	0.83	26
			DG	0.60	0.64	0.62	64
			MNG	0.88	0.40	0.55	55
			Micro Avg (Accuracy)	0.57	0.57	0.57	153
			Macro Avg	0.59	0.53	0.52	153
			Weighted Avg	0.71	0.57	0.60	153

Avg: average, DG: diffuse goiter, MNG: multi-nodular goiter, RFE: recursive feature elimination, MLP: multilayer perceptron

**FIGURE 8:**
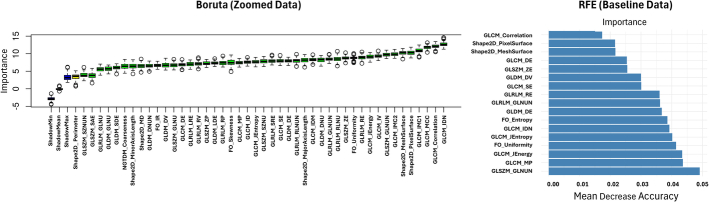
Radiomic features selected by Boruta and RFE feature selection methods when models were trained on zoomed and baseline data sets using mutually robust features. GLCM indicates gray-level co-occurrence matrix; GLDM, gray-level dependence matrix; GLRLM, gray-level run length matrix; GLSZM, gray-level size zone matrix; NGTDM, neighborhood gray-tone difference matrix.

Figure 8 shows the selected robust features by the Boruta and RFE methods. RFE is a backward selection algorithm that iteratively removes the least important features from a model based on a predefined importance metric. In this study, feature importance is quantified using mean decrease accuracy (MDA). MDA reflects the importance of a feature by randomly permuting it and measuring the resulting drop in the model’s predictive accuracy. Therefore, features can be ranked based on their MDA score, where a higher MDA indicates greater importance.^40^ Both feature selection methods agree that the majority of the top 5 most important features are from GLCM. Boruta selected 4 and RFE 3 GLCM features among the top 5.

## DISCUSSION

Radiomics is an approach to medical image analysis that makes it possible to analyze images through quantitative feature extraction.^22^ Several studies have demonstrated that coupling ML and radiomics unlocks objective and experience-independent diagnosis within short times with expert-level accuracy.^41–43^ However, the reproducibility of such features is still under extensive study.^44,45^

To date, limited work has been done on radiomic feature reproducibility in thyroid scintigraphy, and this makes our study unique to the best of our knowledge. Most thyroid studies investigating the predictive power of radiomic-based models or feature reproducibility have used CT, MRI, and PET images.^8,46–49^ Therefore, in the present study, we investigated the impact of intraobserver segmentation variability and zooming on radiomic features’ reproducibility in the context of thyroid scintigraphy. In nuclear thyroid scans, it is a challenging task to differentiate normal and abnormal thyroid specifying the complications (thyroiditis, DG, MNG). Accordingly, in the second step, we employed 7 ML algorithms and 3 feature selection methods to explore the efficacy of robust features under segmentation and zoom variations in enhancing machine performance.

The results of reproducibility assessments showed that the majority (96%) of radiomic features are robust (ICC≥0.80) under intraobserver segmentation variability, irrespective of the zooming condition. The only texture feature that showed excellent robustness (ICC≥0.90) across all batches appeared to be RLNU from GLRLM. In a study by Gharibi et al,^28^ GLRLM was also shown to be robust (ICC≥0.90) under various changes in filter type, filter cutoff, filter order, and even image reconstruction algorithm. Moreover, all the mutually robust features we found in the present study, except for IMC2 and JEnergy from GLCM, LDE from GLDM, and RV from GLRLM, were shown to have ICC≥0.80 in their study. This suggests that GLRLM and most of the features we found mutually robust are reproducible in single-photon emission imaging. However, more investigation into their correlation with diseases is still needed. Reproducibility may not directly provide any clinical advantage of radiomic features, but it is significantly important in helping researchers preselecting features for further analysis in machine learning studies, trust the correlation of a feature to a disease more confidently, and make future research more clinically reliable.^28^ The reported satisfactory ICCs in intra/interobserver segmentation agreement suggest that manual segmentation has the least impact on radiomics reliability, but introduces intraobserver variability. Therefore, using automated segmentation combined with robust features minimizes the impact of intraobserver differences, thus enhancing the reliability of radiomics-based AI applications.^50,51^

In a study by Huang et al,^46^ reproducibility of thyroid CT radiomic features against interobserver and intraobserver segmentation variations was investigated. They found that elongation from shape; IDN, IV, and DE from GLCM; RLNU, LRLGLE, SRE, and RP from GLRLM; SAE and SZNUN from GLSZM; and Coarseness from NGTDM were highly robust, showing ICC≥0.90 under both interobserver and intraobserver segmentation variations. This is consistent with our findings except for IDN (0.80≤ICC˂0.90) and elongation (0.50≤ICC˂0.80). In our study, most of the sensitive features under the segmentation batch were from the shape family. This is while only 49% of the features were reproducible under different zooming. The shape family was the most reproducible under the zoom batch. This finding seems reasonable, as the only disturbing factor affecting the shape of the region of interest (ROI) is the difference in the segmentation layer.

Furthermore, it is evident that most of the features achieving high importance scores were from texture families. This finding is aligned with the results presented by Sabouri et al^52^ and Huang et al.^46^ In their study, Sabouri et al^52^ used single-center thyroid scintigraphy images to train ML models for normal/abnormal classification of cases. They selected the most predictive features iteratively by the MRMR method in each iteration of nested cross-validation. In their study, the top 5 selected features were Coarseness and Strength from NGTDM, M2DDC from Shape, IMC2 from GLCM, and GLV from GLSZM. Instead, IDN, Correlation, MCC, and IMC1 from GLCM, and Pixel Surface from Shape appeared to be the 5 most important features in zoomed data in our study. In a different approach, Huang et al^46^ used the LASSO method to rank the important features extracted from thyroid CT scans and reported coarseness from NGTDM; and RP and SRE from GLRLM as important features, with coarseness being at least twice as important. However, little difference was found between the Boruta importance score of these features in the present study. These combinations probably attained enhanced performance owing to the synergy between the feature selection technique and the model architecture. Boruta and RFE reduce dimensionality by selecting only the most predictive, thereby reducing overfitting and improving model generalization.^53^ MLP models benefit from specialized feature sets due to their complex internal architecture. We found that combining these effective feature selectors with a flexible, nonlinear model, like MLP, improved classification accuracy and robustness by learning from the most informative features.

In another study similar to ours, Sabouri et al^4^ incorporated a larger population, including 2643 patients from 9 medical centers and classified them into different pathologies, including MNG, thyroiditis, and DG. This study aimed to develop an automated pipeline that enhances thyroid disease classification using thyroid scintigraphy images, aiming to decrease assessment time and increase diagnostic accuracy in 2 scenarios. Radiomic features were extracted from both physician (scenario 1) and ResUNet segmentations (scenario 2). ResUNet achieved DSC of 0.84±0.03, 0.71±0.06, and 0.86±0.02 for MNG, TH, and DG, respectively. They selected the most important features for model training in each iteration (9 iterations in total) in a leave-one-center-out cross-validation scheme using RFE after removing highly correlated features with a high Spearman correlation coefficient. Kurtosis and Skewness from FO; DNU and DNUN from GLDM; and Coarseness, Contrast, and Complexity from NGTDM were each selected at least 7 times, reflecting their reproducibility. Classification in scenario 1 achieved an accuracy of 0.76±0.04 and a ROC AUC of 0.92±0.02, while in scenario 2, classification yielded an accuracy of 0.74±0.05 and a ROC AUC of 0.90±0.02. This highlights their consistent relevance across various centers and data types. Except for Kurtosis, Contrast, and Complexity, all other features identified as important were robust against Segmentation and zoom batches. Moreover, DNU and Skewness were ranked relatively important by Boruta. Still, they do not include any normal cases while performing classification.

Turning to the impact of batches on classification performance, another highlight of this study is that the ML models performed generally better in classifying normal and abnormal thyroid patients when trained with only robust radiomic features. This is probably because some features may show high correlation with the disease in the training set, although being irreproducible under a specific batch. Therefore, they will not offer a reliable explanation of the test/validation sets with different imaging conditions and/or parameters. Consequently, poor machine performance can be expected when feature selection is only based on the correlation of a radiomic feature with the disease, overlooking its reproducibility. Also, our results show that models had an easier task identifying thyroiditis (AUC >0.90) compared with other classes. This is while models showed significantly lower performance in differentiating normal cases (AUC: 0.52 and 0.54). This finding of our study is aligned with what Cama et al^47^ presented in a paper exploring the impact of segmentation variations on radiomic features’ robustness and ML performance in predicting breast cancer subtypes. In their study, Cama and colleagues used 3 segmentation masks on breast MRI images and found that feeding ML algorithms with features that are highly reproducible under segmentation variations enhances machine accuracy.

Photopenic defects and discordant nodules pose challenges for accurate thyroid segmentation. In this study, we used a traditional/manual approach to maintain consistency across batches. While our study did not specifically focus on these cases, deep learning-based methods may offer improved consistency by learning complex and contextual patterns, including atypical uptake. Their performance in such challenging scenarios remains an area for future investigation.

In the previously mentioned study by Sabouri et al,^52^ the DT classifier achieved the highest performance using the most predictive combination of 6 features (AUC: 0.81, ACC: 0.78, F1-score: 0.80). In contrast, when utilizing the 10 most predictive features, the RF classifier performed the best (AUC: 0.77, ACC: 0.79, F1-score: 0.83). In the other study by Sabouri et al,^4^ Residual UNet (ResUNet) model was developed for thyroid auto-segmentation to compare the performance of an XGB model in classifying MNG, Thyroiditis, and DG patients when it is fed with physician-segmented and ResUNet-segmented images. They found negligible inferior performance when feeding the model with ResUNet-segmented images (AUC: 0.92 vs. 0.90, ACC: 0.76 vs. 0.74). Such findings, alongside the results of the present study, demonstrate the capability of AI and radiomics in binary and multiclass thyroid disease classification. Also, deep learning segmentation methods can reduce assessment time while maintaining high diagnostic accuracy.

This work involved multiple limitations, including utilizing a single physician investigating intraobserver segmentation variability, although other physicians may be employed for comparative analysis of patient outcomes. Furthermore, the sex predilection differences in centers A and B likely reflect referral patterns, demographics, or health care–seeking behavior, rather than thyroid disease prevalence in the general population. The use of a single-center training dataset may introduce biases related to differences in patient demographics, imaging protocols, and equipment, which could affect the generalizability of the model. Future works should focus on using multicenter training data sets.^54,55^ The baseline training (×1.25) and external validation (×1.78) sets were not precisely in agreement regarding the zoom settings, as we were not able to access other data sets. Moreover, deep learning models ought to be employed on a greater number of patients.

## CONCLUSIONS

Our study demonstrated that most of the radiomic features are reproducible under intraobserver segmentation variabilities. However, zooming can significantly affect >50% of the features. Utilizing reliable radiomic features can significantly enhance the generalizability of ML models. This improvement in generalizability allows the models to perform effectively across diverse data sets and scenarios, ultimately leading to more accurate predictions and better overall outcomes.

## Supplementary Material
