# Supplementary material for: Impact of Field-of-view Zooming and Segmentation Batches on Radiomics Features Reproducibility and Machine Learning Performance in Thyroid Scintigraphy
Source: Clin Nucl Med. 2025 Jun 17;50(8):683–94. doi: 10.1097/RLU.0000000000005995 (PMC12208387; doi:10.1097/RLU.0000000000005995)
Supplement: Supplementary file 1 [file rlu-50-683-s001.pdf]

Table S1 . All features used in this study.

Family	#	Feature name(Abbreviation)	Family	#	Feature name(Abbreviation)				
Gray-Level Run Length Matrix (GLRLM)	1	Gray Level Non-Uniformity (GLNU)	Gray-Level Size Zone Matrix (GLSZM)	1	Gray Level Non-Uniformity (GLNU)				
	2	Gray Level Non-Uniformity Normalized (GLNUN)		2	Gray Level Non-Uniformity Normalized (GLNUN)				
	3	Gray Level Variance (GLV)		3	Gray Level Variance (GLV)				
	4	High Gray Level Run Emphasis (HGLRE)		4	High Gray Level Zone Emphasis (HGLZE)				
	5	Long Run Emphasis (LRE)		5	Large Area Emphasis (LAE)				
	6	Long Run High Gray Level Emphasis (LRHGLE)		6	Large Area High Gray Level Emphasis (LAHGLE)				
	7	Long Run Low Gray Level Emphasis (LRLGLE)		7	Large Area Low Gray Level Emphasis (LALGLE)				
	8	Low Gray Level Run Emphasis (LGLRE)		8	Low Gray Level Zone Emphasis (LGLZE)				
	9	Run Entropy (RE)		9	Size Zone Non-Uniformity (SZNU)				
	10	Run Length Non-Uniformity (RLNU)		10	Size Zone Non-Uniformity Normalized (SZNUN)				
	11	Run Length Non-Uniformity Normalized (RLNUN)		11	Small Area Emphasis (LAE)				
	12	Run Percentage (RP)		12	Small Area High Gray Level Emphasis (SAHGLE)				
	13	Run Variance (RV)		13	Small Area Low Gray Level Emphasis (SALGLE)				
	14	Short Run Emphasis (SRE)		14	Zone Entropy (ZE)				
	15	Short Run High Gray Level Emphasis (SRHGLE)		15	Zone Percentage (ZP)				
	16	Short Run Low Gray Level Emphasis (SRLGLE)		16	Zone Variance (ZV)				
First Order (FO)	1	10th Percentile	Gray-Level Cooccurrence Matrix (GLCM)	1	Autocorrelation				
	2	90th Percentile		2	Cluster Prominence (CP)				
	3	Energy		3	Cluster Shade (CS)				
	4	Entropy		4	Cluster Tendency (CT)				
	5	Interquartile Range (IQR)		5	Contrast				
	6	Kurtosis		6	Correlation				
	7	Maximum		7	Difference Average (DA)				
	8	Mean Absolute Deviation (MAD)		8	Difference Entropy (DE)				
	9	Mean		9	Difference Variance (DV)				
	10	Median		10	Inverse Difference (ID)				
	11	Minimum		11	Inverse Difference Moment (IDM)				
	12	Range		12	Inverse Difference Moment Normalized (IDMN)				
	13	Robust Mean Absolute Deviation (RMAD)		13	Inverse Difference Normalized (IDN)				
	14	Root Mean Squared (RMS)		14	Informational Measure of Correlation 1 (IMC1)				
	15	Skewness		15	Informational Measure of Correlation 2 (IMC2)				
	16	Total Energy (TE)		16	Inverse Variance (IV)				
	17	Uniformity		17	Joint Average (JA)				
	18	Variance		18	Joint Energy (JEnergy)				
Gray-Level Dependence Matrix (GLDM)	1	Dependence Entropy (DE)		19	Joint Entropy (JEntropy)				
	2	Dependence Non-Uniformity (DNU)		20	Maximal Correlation Coefficient (MCC)				
	3	Dependence Non-Uniformity Normalized (DNUN)		21	Maximum Probability (MP)				
	4	Dependence Variance (DV)		22	Sum Average (SA)				
	5	Gray Level Non-Uniformity (GLNU)		23	Sum Entropy (SE)				
	6	Gray Level Variance (GLV)		24	Sum of Squares (SS)				
	7	High Gray Level Emphasis (HGLE)	Neighborhood Gray-Tone Difference	1	Busyness				
	8	Large Dependence Emphasis (LDE)		2	Coarseness				
	9	Large Dependence High Gray Level Emphasis (LDHGLE)		3	Complexity				
	10	Large Dependence Low Gray Level Emphasis (LDLGLE)		4	Contrast				
	11	Low Gray Level Emphasis (LGLE)		5	Strength				
	12	Small Dependence Emphasis (SDE)	Shape	1	MeshSurface	4	MajorAxisLength	7	Elongation
	13	Small Dependence High Gray Level Emphasis (SDHGLE)		2	Perimeter	5	Sphericity	8	MaximumDiameter
	14	Small Dependence Low Gray Level Emphasis (SDLGLE)		3	MinorAxisLength	6	PerimeterSurfaceRatio	9	PixelSurfaArea

Table S2. ICC scores of features under segmentation batch in zoomed dataset (Segment_Z).

Feature Family	ICC < 0.80	0.80 ≤ ICC < 0.90	0.90 ≤ ICC ≤ 1.00
Shape	PerimeterSurfaceRatio, Sphericity, Elongation	MeshSurface, PixelSurface MinorAxisLength MaximumDiameter	Perimeter, MajorAxisLength
FO	Minimum	10Percentile, Kurtosis, Skewness	Maximum, Energy, TE, Range, Median, RMS, RMAD, MAD, Variance, Entropy, 90Percentile, Mean, IQR, Uniformity
GLCM		IDN	IDMN, MP, CS, SA, JA, DE, JEntropy, SS, CT, IV, IMC1, ID, IDM, MCC, DV, Autocorrelation, Correlation, , CP, SE, IMC2, Contrast, DA, JEnergy
GLDM		SDLGLE, DV	LGLE, LDLGLE, GLNU, DNU, HGLE, DNUN, SDHGLE, DE, LDHGLE, LDE, GLV, SDE
GLRLM			LRLGLE, SRLGLE, LGLRE, RLNU, GLNU, LRE, RV, SRHGLE, HGLRE, LRHGLE, RP, SRE, GLNUN, RLNUN, RE, GLV
GLSZM			LALGLE, SALGLE, ZV, LAE, LGLZE, LAHGLE, GLNU, ZE, SZNU, SAE, HGLZE, ZP, GLV, GLNUN, SAHGLE
NGTDM			Coarseness, Busyness, Contrast, Complexity, Strength

Table S3. ICC scores of features under segmentation batch in baseline dataset (Segment_WZ).

Feature Family	ICC < 0.80	0.80 ≤ ICC < 0.90	0.90 ≤ ICC ≤ 1.00
Shape	PerimeterSurfaceRatio, Sphericity, Elongation,	MaximumDiameter, MeshSurface, PixelSurface, MinorAxisLength, MajorAxisLength,	Perimeter
FO	Kurtosis	10Percentile, Minimum	Maximum, Energy, TE, Range, Median, RMS, RMAD, MAD, Variance, Entropy, 90Percentile, Mean, IQR, Uniformity, Skewness
GLCM			IDN, IDMN, MP, CS, SA, JA, DE, JEntropy, SS, CT, IV, IMC1, ID, IDM, MCC, DV, Autocorrelation, Correlation, CP, SE, IMC2, Contrast, DA, JEnergy
GLDM		SDLGLE	LGLE, LDLGLE, GLNU, DNU, HGLE, DNUN, SDHGLE, DE, LDHGLE, LDE, GLV, SDE , DV
GLRLM			LRLGLE, SRLGLE, LGLRE, RLNU, GLNU, LRE, RV, SRHGLE, HGLRE, LRHGLE, RP, SRE, GLNUN, RLNUN, RE, GLV
GLSZM		SALGLE	LALGLE, , ZV, LAE, LGLZE, LAHGLE, GLNU, ZE, SZNU, SAE, HGLZE, ZP, GLV, GLNUN, SAHGLE
NGTDM			Coarseness, Busyness, Contrast, Complexity, Strength

Table S4. ICC scores of features under zoom batch (Zoom).

Feature Family	ICC < 0.80	0.80 ≤ ICC < 0.90	0.90 ≤ ICC ≤ 1.00
Shape		PerimeterSurfaceRatio, Sphericity, Elongation,	MaximumDiameter, MeshSurface, PixelSurface, MinorAxisLength, MajorAxisLength, Perimeter
FO	Maximum, Energy, TE, Range, MAD, Variance, RMS, 90Percentile, RMAD, IQR, Mean, Median, Minimum, 10Percentile,	Entropy, Uniformity,	Kurtosis, Skewness
GLCM	IDMN, CS, SA, JA, SS, CT, DV, Autocorrelation, CP, Contrast, DA	IDN, MP, DE, JEntropy, IV, IMC1, ID, IDM, MCC, Correlation, SE, IMC2, JEnergy	
GLDM	GLV, SDHGLE, HGLE, LDLGLE, LDHGLE, LGLE, SDLGLE,	DV, DNU, DNUN, SDE, DE, GLNU, LDE	
GLRLM	GLV, SRHGLE, HGLRE, LRHGLE, LGLRE, SRLGLE, LRLGLE	GLNUN, RLNUN, GLNU, RV, LRE, RP, SRE, RE,	RLNU
GLSZM	GLV, SAHGLE, HGLZE, LAHGLE, LAE, LGLZE, ZV, LALGLE, SALGLE	GLNUN, SZNU,GLNU, ZE, ZP, SAE, SZNUN	
NGTDM	Strength, Complexity, Contrast, Busyness	Coarseness,	

Table S5. Comparison of performance between all the models trained using all features extracted from Baseline dataset and tested on Baseline etxternal dataset.

		AUC			Precision			Recall			F1-score		
Model	Accuracy	macro	micro	weighted	macro	micro	weighted	macro	micro	weighted	macro	micro	weighted
RFE_MLP	0.60	0.76	0.73	0.75	0.64	0.60	0.69	0.65	0.60	0.60	0.60	0.60	0.59
Boruta_XGB	0.59	0.83	0.80	0.81	0.64	0.59	0.66	0.65	0.59	0.59	0.60	0.59	0.58
RFE_NB	0.59	0.86	0.83	0.86	0.63	0.59	0.67	0.63	0.59	0.59	0.59	0.59	0.58
RFE_SVM	0.59	0.87	0.83	0.87	0.67	0.59	0.72	0.65	0.59	0.59	0.59	0.59	0.57
RFE_KNN	0.57	0.82	0.80	0.82	0.64	0.57	0.69	0.63	0.57	0.57	0.57	0.57	0.56
RFE_RF	0.57	0.86	0.82	0.85	0.66	0.57	0.71	0.64	0.57	0.57	0.57	0.57	0.55
RFE_XGB	0.57	0.84	0.80	0.83	0.63	0.57	0.68	0.63	0.57	0.57	0.55	0.57	0.53
Boruta_RF	0.55	0.81	0.79	0.79	0.60	0.55	0.62	0.61	0.55	0.55	0.56	0.55	0.54
Boruta_KNN	0.54	0.73	0.74	0.71	0.61	0.54	0.62	0.57	0.54	0.54	0.56	0.54	0.54
Boruta_MLP	0.54	0.68	0.69	0.68	0.58	0.54	0.58	0.52	0.54	0.54	0.53	0.54	0.54
RFE_DT	0.55	0.76	0.77	0.74	0.64	0.55	0.70	0.61	0.55	0.55	0.54	0.55	0.53
Boruta_DT	0.53	0.80	0.77	0.78	0.60	0.53	0.61	0.60	0.53	0.53	0.55	0.53	0.52
MRMR_MLP	0.54	0.70	0.69	0.69	0.55	0.54	0.57	0.56	0.54	0.54	0.54	0.54	0.54
MRMR_RF	0.52	0.78	0.75	0.76	0.58	0.52	0.58	0.58	0.52	0.52	0.54	0.52	0.51
MRMR_XGB	0.51	0.77	0.74	0.76	0.60	0.51	0.62	0.54	0.51	0.51	0.52	0.51	0.51
Boruta_NB	0.50	0.75	0.73	0.75	0.50	0.50	0.51	0.50	0.50	0.50	0.49	0.5	0.49
MRMR_NB	0.49	0.80	0.77	0.79	0.55	0.49	0.55	0.50	0.49	0.49	0.50	0.49	0.50
MRMR_KNN	0.48	0.72	0.73	0.70	0.55	0.48	0.56	0.51	0.48	0.48	0.50	0.48	0.49
MRMR_SVM	0.44	0.67	0.67	0.66	0.55	0.44	0.56	0.46	0.44	0.44	0.46	0.44	0.46
MRMR_DT	0.40	0.71	0.68	0.69	0.41	0.40	0.38	0.48	0.40	0.40	0.40	0.40	0.34
Boruta_SVM	0.20	0.68	0.52	0.67	0.34	0.20	0.43	0.26	0.20	0.20	0.14	0.20	0.13

RFE: recursive feature elimination, MRMR: minimum redundancy maximum relevance, MLP: multi-layer perceptron, DT: decision tree, KNN: k-nearest neighbors, NB: naïve bayes, RF: random forest, SVM: support vector machine, XGB: extreme gradient boosting.

Table S6. Comparison of performance between all the models trained using all features extracted from Baseline dataset and tested on Zoomed etxternal dataset.

		AUC			Precision			Recall			F1-score		
Model	Accuracy	macro	micro	weighted	macro	micro	weighted	macro	micro	weighted	macro	micro	weighted
MRMR_XGB	0.49	0.78	0.79	0.82	0.51	0.49	0.69	0.48	0.49	0.49	0.41	0.49	0.50
Boruta_XGB	0.34	0.78	0.69	0.80	0.47	0.34	0.65	0.41	0.34	0.34	0.30	0.34	0.34
MRMR_RF	0.41	0.73	0.71	0.75	0.55	0.41	0.74	0.51	0.41	0.41	0.38	0.41	0.42
Boruta_RF	0.32	0.73	0.63	0.72	0.53	0.32	0.72	0.49	0.32	0.32	0.29	0.32	0.28
RFE_XGB	0.21	0.72	0.61	0.76	0.19	0.21	0.24	0.28	0.21	0.21	0.13	0.21	0.13
MRMR_NB	0.30	0.70	0.63	0.72	0.53	0.30	0.74	0.44	0.30	0.30	0.28	0.30	0.28
Boruta_KNN	0.31	0.70	0.63	0.72	0.46	0.31	0.62	0.42	0.31	0.31	0.28	0.31	0.30
Boruta_NB	0.26	0.69	0.63	0.70	0.42	0.26	0.55	0.44	0.26	0.26	0.24	0.26	0.21
MRMR_DT	0.54	0.68	0.75	0.68	0.44	0.54	0.60	0.48	0.54	0.54	0.40	0.54	0.51
MRMR_KNN	0.29	0.66	0.58	0.66	0.36	0.29	0.48	0.40	0.29	0.29	0.26	0.29	0.26
Boruta_DT	0.32	0.65	0.65	0.63	0.51	0.32	0.70	0.49	0.32	0.32	0.29	0.32	0.26
Boruta_SVM	0.38	0.62	0.72	0.67	0.34	0.38	0.55	0.26	0.38	0.38	0.16	0.38	0.24
Boruta_MLP	0.21	0.61	0.47	0.57	0.38	0.21	0.44	0.44	0.21	0.21	0.20	0.21	0.13
RFE_KNN	0.27	0.60	0.52	0.62	0.21	0.27	0.26	0.32	0.27	0.27	0.19	0.27	0.20
MRMR_SVM	0.33	0.58	0.71	0.62	0.24	0.33	0.38	0.22	0.33	0.33	0.22	0.33	0.34
RFE_RF	0.23	0.57	0.45	0.55	0.18	0.23	0.23	0.29	0.23	0.23	0.15	0.23	0.15
RFE_DT	0.23	0.54	0.43	0.54	0.20	0.23	0.25	0.29	0.23	0.23	0.15	0.23	0.15
RFE_MLP	0.21	0.52	0.47	0.52	0.19	0.21	0.24	0.28	0.21	0.21	0.13	0.21	0.13
MRMR_MLP	0.20	0.51	0.46	0.53	0.50	0.20	0.73	0.27	0.20	0.20	0.13	0.20	0.12
RFE_NB	0.27	0.49	0.55	0.56	0.31	0.27	0.45	0.25	0.27	0.27	0.22	0.27	0.28
RFE_SVM	0.28	0.49	0.48	0.51	0.20	0.28	0.25	0.32	0.28	0.28	0.19	0.28	0.21

RFE: recursive feature elimination, MRMR: minimum redundancy maximum relevance, MLP: multi-layer perceptron, DT: decision tree, KNN: k-nearest neighbors, NB: naïve bayes, RF: random forest, SVM: support vector machine, XGB: extreme gradient boosting.

Table S7. Comparison of performance between all the models trained using all features extracted from Zoomed dataset and tested on Zoomed etxternal dataset.

		AUC			Precision			Recall			F1-score		
Model	Accuracy	macro	micro	weighted	macro	micro	weighted	macro	micro	weighted	macro	micro	weighted
MRMR_NB	0.63	0.81	0.84	0.84	0.58	0.63	0.74	0.59	0.63	0.63	0.54	0.63	0.64
MRMR_XGB	0.63	0.86	0.87	0.88	0.61	0.63	0.75	0.65	0.63	0.63	0.58	0.63	0.65
RFE_NB	0.62	0.81	0.85	0.83	0.58	0.62	0.71	0.64	0.62	0.62	0.57	0.62	0.64
RFE_SVM	0.59	0.84	0.85	0.85	0.60	0.59	0.73	0.65	0.59	0.59	0.56	0.59	0.61
MRMR_RF	0.57	0.85	0.85	0.86	0.60	0.57	0.76	0.64	0.57	0.57	0.53	0.57	0.57
Boruta_MLP	0.56	0.69	0.71	0.70	0.58	0.56	0.71	0.53	0.56	0.56	0.52	0.56	0.60
RFE_KNN	0.56	0.81	0.83	0.83	0.57	0.56	0.70	0.58	0.56	0.56	0.52	0.56	0.58
RFE_XGB	0.54	0.85	0.86	0.86	0.57	0.54	0.70	0.59	0.54	0.54	0.51	0.54	0.55
RFE_RF	0.53	0.83	0.84	0.84	0.56	0.53	0.70	0.58	0.53	0.53	0.49	0.53	0.53
Boruta_DT	0.52	0.79	0.80	0.80	0.59	0.52	0.74	0.58	0.52	0.52	0.49	0.52	0.53
MRMR_SVM	0.51	0.80	0.81	0.81	0.49	0.51	0.63	0.43	0.51	0.51	0.41	0.51	0.50
RFE_DT	0.55	0.78	0.83	0.78	0.56	0.55	0.69	0.60	0.55	0.55	0.53	0.55	0.58
RFE_MLP	0.52	0.73	0.68	0.71	0.58	0.52	0.71	0.60	0.52	0.52	0.51	0.52	0.56
Boruta_XGB	0.50	0.82	0.81	0.83	0.57	0.50	0.72	0.60	0.50	0.50	0.49	0.50	0.53
MRMR_DT	0.50	0.81	0.81	0.81	0.60	0.50	0.75	0.62	0.50	0.50	0.51	0.50	0.55
Boruta_NB	0.50	0.81	0.81	0.83	0.59	0.50	0.75	0.59	0.50	0.50	0.49	0.50	0.54
MRMR_KNN	0.49	0.81	0.82	0.81	0.50	0.49	0.62	0.55	0.49	0.49	0.47	0.49	0.51
Boruta_KNN	0.45	0.77	0.76	0.77	0.48	0.45	0.60	0.54	0.45	0.45	0.43	0.45	0.47
Boruta_RF	0.45	0.81	0.77	0.80	0.57	0.45	0.72	0.56	0.45	0.45	0.46	0.45	0.49
MRMR_MLP	0.40	0.67	0.60	0.65	0.57	0.40	0.75	0.52	0.40	0.40	0.35	0.40	0.38
Boruta_SVM	0.38	0.74	0.75	0.76	0.34	0.38	0.55	0.26	0.38	0.38	0.16	0.38	0.24

RFE: recursive feature elimination, MRMR: minimum redundancy maximum relevance, MLP: multi-layer perceptron, DT: decision tree, KNN: k-nearest neighbors, NB: naïve bayes, RF: random forest, SVM: support vector machine, XGB: extreme gradient boosting.

Table S8. Comparison of performance between all the models trained using all features extracted from Zoomed dataset and tested on Baseline etxternal dataset.

		AUC			Precision			Recall			F1-score		
Model	Accuracy	macro	micro	weighted	macro	micro	weighted	macro	micro	weighted	macro	micro	weighted
RFE_NB	0.36	0.78	0.69	0.76	0.41	0.36	0.35	0.41	0.36	0.36	0.36	0.36	0.31
Boruta_XGB	0.41	0.74	0.67	0.72	0.50	0.41	0.49	0.47	0.41	0.41	0.42	0.41	0.38
MRMR_RF	0.38	0.74	0.63	0.71	0.50	0.38	0.49	0.45	0.38	0.38	0.38	0.38	0.34
Boruta_DT	0.39	0.73	0.65	0.71	0.47	0.39	0.44	0.45	0.39	0.39	0.39	0.39	0.35
RFE_RF	0.35	0.72	0.65	0.69	0.43	0.35	0.39	0.42	0.35	0.35	0.35	0.35	0.31
Boruta_RF	0.39	0.72	0.64	0.69	0.49	0.39	0.47	0.44	0.39	0.39	0.39	0.39	0.36
RFE_XGB	0.37	0.71	0.65	0.67	0.45	0.37	0.41	0.44	0.37	0.37	0.38	0.37	0.33
RFE_KNN	0.32	0.69	0.64	0.66	0.42	0.32	0.39	0.39	0.32	0.32	0.34	0.32	0.29
MRMR_XGB	0.40	0.68	0.64	0.65	0.52	0.40	0.51	0.45	0.40	0.40	0.39	0.40	0.35
RFE_DT	0.38	0.68	0.63	0.65	0.47	0.38	0.43	0.42	0.38	0.38	0.40	0.38	0.36
MRMR_DT	0.41	0.68	0.66	0.67	0.51	0.41	0.49	0.42	0.41	0.41	0.40	0.41	0.38
MRMR_NB	0.30	0.64	0.58	0.62	0.37	0.30	0.33	0.33	0.30	0.30	0.31	0.30	0.28
MRMR_KNN	0.35	0.64	0.60	0.61	0.42	0.35	0.38	0.38	0.35	0.35	0.31	0.35	0.28
RFE_SVM	0.27	0.62	0.58	0.59	0.37	0.27	0.33	0.35	0.27	0.27	0.28	0.27	0.23
Boruta_KNN	0.23	0.60	0.59	0.59	0.39	0.23	0.37	0.28	0.23	0.23	0.26	0.23	0.23
RFE_MLP	0.36	0.59	0.57	0.57	0.48	0.36	0.46	0.39	0.36	0.36	0.36	0.36	0.34
Boruta_SVM	0.15	0.54	0.42	0.52	0.04	0.15	0.02	0.24	0.15	0.15	0.07	0.15	0.04
MRMR_MLP	0.27	0.52	0.51	0.51	0.36	0.27	0.34	0.29	0.27	0.27	0.26	0.27	0.24
Boruta_NB	0.18	0.50	0.46	0.49	0.19	0.18	0.18	0.20	0.18	0.18	0.18	0.18	0.17
MRMR_SVM	0.22	0.49	0.44	0.45	0.31	0.22	0.26	0.31	0.22	0.22	0.22	0.22	0.17
Boruta_MLP	0.20	0.49	0.47	0.48	0.40	0.20	0.38	0.24	0.20	0.20	0.21	0.20	0.19

RFE: recursive feature elimination, MRMR: minimum redundancy maximum relevance, MLP: multi-layer perceptron, DT: decision tree, KNN: k-nearest neighbors, NB: naïve bayes, RF: random forest, SVM: support vector machine, XGB: extreme gradient boosting.

Table S9. Comparison of performance between all the models trained using robust features against zoom batch extracted from Baseline dataset and tested on Zoomed etxternal dataset.

		AUC			Precision			Recall			F1-score		
Model	Accuracy	macro	micro	weighted	macro	micro	weighted	macro	micro	weighted	macro	micro	weighted
MRMR_NB	0.55	0.80	0.80	0.83	0.63	0.55	0.82	0.63	0.55	0.55	0.53	0.55	0.60
MRMR_XGB	0.41	0.78	0.75	0.79	0.51	0.41	0.67	0.54	0.41	0.41	0.39	0.41	0.41
Boruta_XGB	0.39	0.78	0.73	0.79	0.52	0.39	0.70	0.53	0.39	0.39	0.36	0.39	0.38
MRMR_RF	0.39	0.78	0.74	0.79	0.54	0.39	0.73	0.53	0.39	0.39	0.36	0.39	0.37
Boruta_NB	0.49	0.77	0.75	0.78	0.54	0.49	0.69	0.59	0.49	0.49	0.47	0.49	0.50
MRMR_KNN	0.45	0.76	0.73	0.76	0.52	0.45	0.67	0.54	0.45	0.45	0.42	0.45	0.47
Boruta_KNN	0.43	0.75	0.71	0.76	0.50	0.43	0.66	0.53	0.43	0.43	0.40	0.43	0.44
Boruta_RF	0.37	0.75	0.70	0.75	0.53	0.37	0.72	0.51	0.37	0.37	0.34	0.37	0.35
MRMR_SVM	0.45	0.71	0.76	0.74	0.41	0.45	0.57	0.42	0.45	0.45	0.37	0.45	0.48
Boruta_SVM	0.38	0.69	0.73	0.74	0.34	0.38	0.55	0.26	0.38	0.38	0.16	0.38	0.24
RFE_RF	0.26	0.69	0.54	0.73	0.23	0.26	0.27	0.34	0.26	0.26	0.19	0.26	0.19
RFE_XGB	0.24	0.69	0.57	0.72	0.28	0.24	0.34	0.35	0.24	0.24	0.19	0.24	0.18
RFE_NB	0.25	0.69	0.56	0.72	0.26	0.25	0.32	0.33	0.25	0.25	0.19	0.25	0.19
Boruta_DT	0.32	0.68	0.68	0.68	0.26	0.32	0.28	0.48	0.32	0.32	0.28	0.32	0.25
MRMR_DT	0.30	0.68	0.66	0.68	0.27	0.30	0.29	0.47	0.30	0.30	0.27	0.30	0.24
RFE_KNN	0.27	0.68	0.55	0.71	0.25	0.27	0.31	0.34	0.27	0.27	0.21	0.27	0.21
RFE_DT	0.31	0.67	0.67	0.69	0.28	0.31	0.32	0.45	0.31	0.31	0.27	0.31	0.25
MRMR_MLP	0.28	0.61	0.52	0.59	0.60	0.28	0.84	0.42	0.28	0.28	0.25	0.28	0.24
RFE_MLP	0.25	0.57	0.50	0.56	0.30	0.25	0.37	0.36	0.25	0.25	0.20	0.25	0.19
Boruta_MLP	0.22	0.56	0.48	0.53	0.28	0.22	0.35	0.36	0.22	0.22	0.18	0.22	0.14
RFE_SVM	0.24	0.45	0.41	0.44	0.32	0.24	0.40	0.35	0.24	0.24	0.19	0.24	0.17

RFE: recursive feature elimination, MRMR: minimum redundancy maximum relevance, MLP: multi-layer perceptron, DT: decision tree, KNN: k-nearest neighbors, NB: naïve bayes, RF: random forest, SVM: support vector machine, XGB: extreme gradient boosting.

Table S10. Comparison of performance between all the models trained using robust features against zoom batch extracted from Zoomed dataset and tested on Zoomed etxternal dataset.

		AUC			Precision			Recall			F1-score		
Model	Accuracy	macro	micro	weighted	macro	micro	weighted	macro	micro	weighted	macro	micro	weighted
RFE_SVM	0.52	0.84	0.81	0.85	0.62	0.52	0.76	0.62	0.52	0.52	0.52	0.52	0.56
MRMR_XGB	0.54	0.84	0.83	0.87	0.59	0.54	0.74	0.65	0.54	0.54	0.50	0.54	0.53
Boruta_XGB	0.57	0.84	0.85	0.86	0.58	0.57	0.71	0.64	0.57	0.57	0.54	0.57	0.59
RFE_XGB	0.56	0.84	0.83	0.86	0.59	0.56	0.73	0.64	0.56	0.56	0.52	0.56	0.55
Boruta_NB	0.66	0.84	0.88	0.85	0.60	0.66	0.69	0.62	0.66	0.66	0.60	0.66	0.67
MRMR_NB	0.66	0.84	0.88	0.87	0.56	0.66	0.69	0.58	0.66	0.66	0.56	0.66	0.67
RFE_RF	0.53	0.83	0.83	0.85	0.56	0.53	0.69	0.59	0.53	0.53	0.49	0.53	0.51
Boruta_RF	0.52	0.82	0.81	0.83	0.59	0.52	0.74	0.64	0.52	0.52	0.5	0.52	0.52
MRMR_RF	0.52	0.82	0.79	0.83	0.58	0.52	0.73	0.62	0.52	0.52	0.49	0.52	0.51
RFE_DT	0.52	0.82	0.82	0.83	0.59	0.52	0.73	0.61	0.52	0.52	0.49	0.52	0.51
RFE_KNN	0.62	0.81	0.82	0.83	0.61	0.62	0.76	0.64	0.62	0.62	0.57	0.62	0.65
MRMR_KNN	0.54	0.80	0.81	0.82	0.51	0.54	0.64	0.57	0.54	0.54	0.49	0.54	0.55
RFE_NB	0.64	0.80	0.83	0.83	0.61	0.64	0.77	0.62	0.64	0.64	0.56	0.64	0.66
Boruta_KNN	0.58	0.80	0.83	0.81	0.52	0.58	0.64	0.57	0.58	0.58	0.51	0.58	0.59
Boruta_SVM	0.35	0.79	0.72	0.81	0.08	0.35	0.12	0.25	0.35	0.35	0.13	0.35	0.19
Boruta_DT	0.52	0.79	0.80	0.80	0.59	0.52	0.74	0.58	0.52	0.52	0.49	0.52	0.53
MRMR_DT	0.50	0.78	0.81	0.79	0.61	0.50	0.77	0.62	0.50	0.50	0.49	0.50	0.53
MRMR_SVM	0.45	0.77	0.76	0.78	0.35	0.45	0.54	0.33	0.45	0.45	0.27	0.45	0.39
RFE_MLP	0.52	0.74	0.68	0.72	0.61	0.52	0.75	0.63	0.52	0.52	0.53	0.52	0.58
MRMR_MLP	0.56	0.72	0.70	0.71	0.58	0.56	0.73	0.59	0.56	0.56	0.50	0.56	0.56
Boruta_MLP	0.66	0.71	0.77	0.73	0.59	0.66	0.68	0.56	0.66	0.66	0.56	0.66	0.64

RFE: recursive feature elimination, MRMR: minimum redundancy maximum relevance, MLP: multi-layer perceptron, DT: decision tree, KNN: k-nearest neighbors, NB: naïve bayes, RF: random forest, SVM: support vector machine, XGB: extreme gradient boosting.

Table S11. Comparison of performance between all the models trained using robust features against zoom batch extracted from Zoomed dataset and tested on Baseline etxternal dataset.

		AUC			Precision			Recall			F1-score		
Model	Accuracy	macro	micro	weighted	macro	micro	weighted	macro	micro	weighted	macro	micro	weighted
RFE_NB	0.67	0.88	0.86	0.88	0.69	0.67	0.72	0.71	0.67	0.67	0.67	0.67	0.66
RFE_RF	0.62	0.84	0.84	0.84	0.67	0.62	0.71	0.67	0.62	0.62	0.62	0.62	0.61
RFE_XGB	0.64	0.84	0.85	0.84	0.63	0.64	0.65	0.65	0.64	0.64	0.64	0.64	0.64
Boruta_XGB	0.61	0.83	0.79	0.82	0.65	0.61	0.67	0.66	0.61	0.61	0.62	0.61	0.59
RFE_SVM	0.64	0.83	0.84	0.82	0.65	0.64	0.68	0.67	0.64	0.64	0.65	0.64	0.65
RFE_KNN	0.63	0.82	0.82	0.81	0.67	0.63	0.71	0.67	0.63	0.63	0.64	0.63	0.63
Boruta_RF	0.56	0.82	0.78	0.80	0.62	0.56	0.64	0.62	0.56	0.56	0.57	0.56	0.55
MRMR_RF	0.54	0.81	0.76	0.79	0.60	0.54	0.62	0.60	0.54	0.54	0.55	0.54	0.52
MRMR_XGB	0.53	0.80	0.75	0.78	0.58	0.53	0.59	0.59	0.53	0.53	0.54	0.53	0.50
MRMR_NB	0.43	0.80	0.74	0.79	0.5	0.43	0.47	0.47	0.43	0.43	0.43	0.43	0.4
RFE_MLP	0.68	0.79	0.78	0.79	0.68	0.68	0.71	0.7	0.68	0.68	0.67	0.68	0.68
Boruta_NB	0.38	0.79	0.72	0.80	0.47	0.38	0.46	0.40	0.38	0.38	0.36	0.38	0.34
RFE_DT	0.55	0.78	0.77	0.77	0.61	0.55	0.64	0.61	0.55	0.55	0.56	0.55	0.55
Boruta_DT	0.55	0.78	0.76	0.77	0.61	0.55	0.63	0.61	0.55	0.55	0.57	0.55	0.55
Boruta_SVM	0.29	0.77	0.62	0.75	0.36	0.29	0.43	0.36	0.29	0.29	0.23	0.29	0.21
Boruta_KNN	0.51	0.77	0.74	0.75	0.56	0.51	0.56	0.54	0.51	0.51	0.51	0.51	0.49
MRMR_DT	0.52	0.77	0.74	0.75	0.57	0.52	0.58	0.57	0.52	0.52	0.53	0.52	0.51
MRMR_KNN	0.50	0.76	0.73	0.74	0.55	0.50	0.54	0.55	0.50	0.50	0.51	0.50	0.47
MRMR_SVM	0.43	0.76	0.69	0.75	0.50	0.43	0.50	0.49	0.43	0.43	0.43	0.43	0.39
MRMR_MLP	0.60	0.74	0.73	0.73	0.62	0.60	0.62	0.61	0.60	0.60	0.60	0.60	0.60
Boruta_MLP	0.53	0.69	0.69	0.69	0.52	0.53	0.60	0.53	0.53	0.53	0.44	0.53	0.46

RFE: recursive feature elimination, MRMR: minimum redundancy maximum relevance, MLP: multi-layer perceptron, DT: decision tree, KNN: k-nearest neighbors, NB: naïve bayes, RF: random forest, SVM: support vector machine, XGB: extreme gradient boosting.

Table S12. Comparison of performance between all the models trained using robust features against zoom batch extracted from Baseline dataset and tested on Baseline etxternal dataset.

		AUC			Precision			Recall			F1-score		
Model	Accuracy	macro	micro	weighted	macro	micro	weighted	macro	micro	weighted	macro	micro	weighted
MRMR_XGB	0.43	0.77	0.71	0.75	0.49	0.43	0.46	0.50	0.43	0.43	0.44	0.43	0.39
RFE_NB	0.39	0.76	0.7	0.74	0.44	0.39	0.39	0.45	0.39	0.39	0.40	0.39	0.35
MRMR_RF	0.39	0.75	0.68	0.73	0.47	0.39	0.44	0.47	0.39	0.39	0.40	0.39	0.36
Boruta_RF	0.39	0.75	0.66	0.72	0.47	0.39	0.44	0.46	0.39	0.39	0.40	0.39	0.36
Boruta_XGB	0.40	0.74	0.69	0.72	0.46	0.40	0.42	0.47	0.40	0.40	0.41	0.40	0.36
MRMR_NB	0.30	0.73	0.63	0.74	0.37	0.30	0.33	0.32	0.30	0.30	0.23	0.30	0.21
Boruta_DT	0.39	0.73	0.65	0.71	0.47	0.39	0.44	0.45	0.39	0.39	0.39	0.39	0.35
RFE_SVM	0.38	0.73	0.66	0.70	0.44	0.38	0.40	0.45	0.38	0.38	0.37	0.38	0.32
MRMR_KNN	0.42	0.72	0.67	0.71	0.50	0.42	0.50	0.46	0.42	0.42	0.41	0.42	0.37
RFE_DT	0.38	0.72	0.65	0.69	0.46	0.38	0.43	0.45	0.38	0.38	0.39	0.38	0.35
RFE_RF	0.32	0.71	0.64	0.68	0.42	0.32	0.39	0.4	0.32	0.32	0.33	0.32	0.29
MRMR_DT	0.42	0.71	0.64	0.69	0.51	0.42	0.50	0.46	0.42	0.42	0.43	0.42	0.40
RFE_KNN	0.35	0.69	0.62	0.66	0.43	0.35	0.39	0.42	0.35	0.35	0.36	0.35	0.32
Boruta_NB	0.25	0.67	0.57	0.67	0.38	0.25	0.35	0.25	0.25	0.25	0.16	0.25	0.16
MRMR_SVM	0.25	0.67	0.57	0.63	0.37	0.25	0.32	0.32	0.25	0.25	0.22	0.25	0.19
RFE_XGB	0.27	0.65	0.61	0.61	0.37	0.27	0.35	0.34	0.27	0.27	0.28	0.27	0.24
RFE_MLP	0.37	0.61	0.58	0.59	0.45	0.37	0.40	0.43	0.37	0.37	0.37	0.37	0.32
Boruta_KNN	0.32	0.61	0.6	0.58	0.40	0.32	0.36	0.35	0.32	0.32	0.31	0.32	0.28
MRMR_MLP	0.40	0.60	0.60	0.59	0.50	0.40	0.51	0.41	0.40	0.40	0.37	0.40	0.36
Boruta_SVM	0.17	0.54	0.46	0.51	0.06	0.17	0.05	0.25	0.17	0.17	0.08	0.17	0.05
Boruta_MLP	0.18	0.47	0.45	0.47	0.58	0.18	0.64	0.20	0.18	0.18	0.15	0.18	0.13

RFE: recursive feature elimination, MRMR: minimum redundancy maximum relevance, MLP: multi-layer perceptron, DT: decision tree, KNN: k-nearest neighbors, NB: naïve bayes, RF: random forest, SVM: support vector machine, XGB: extreme gradient boosting.

Table S13. Comparison of performance between all the models trained using robust features against segmentation batch extracted from Baseline dataset and tested on Zoomed etxternal dataset.

		AUC			Precision			Recall			F1-score		
Model	Accuracy	macro	micro	weighted	macro	micro	weighted	macro	micro	weighted	macro	micro	weighted
MRMR_XGB	0.38	0.78	0.73	0.80	0.49	0.38	0.66	0.5	0.38	0.38	0.35	0.38	0.37
Boruta_XGB	0.36	0.78	0.69	0.80	0.53	0.36	0.74	0.45	0.36	0.36	0.33	0.36	0.36
RFE_XGB	0.34	0.77	0.66	0.78	0.50	0.34	0.69	0.47	0.34	0.34	0.30	0.34	0.30
MRMR_RF	0.41	0.74	0.71	0.75	0.55	0.41	0.74	0.51	0.41	0.41	0.39	0.41	0.43
RFE_NB	0.30	0.73	0.63	0.75	0.50	0.30	0.69	0.42	0.30	0.30	0.27	0.30	0.27
MRMR_NB	0.33	0.72	0.65	0.73	0.51	0.33	0.70	0.49	0.33	0.33	0.31	0.33	0.31
Boruta_RF	0.31	0.72	0.63	0.72	0.52	0.31	0.72	0.48	0.31	0.31	0.28	0.31	0.27
RFE_RF	0.31	0.71	0.57	0.73	0.48	0.31	0.67	0.42	0.31	0.31	0.26	0.31	0.25
RFE_KNN	0.33	0.71	0.64	0.74	0.43	0.33	0.61	0.39	0.33	0.33	0.28	0.33	0.31
MRMR_KNN	0.39	0.70	0.66	0.71	0.45	0.39	0.60	0.46	0.39	0.39	0.34	0.39	0.38
Boruta_KNN	0.30	0.70	0.63	0.72	0.42	0.30	0.56	0.41	0.30	0.30	0.28	0.30	0.28
Boruta_NB	0.26	0.69	0.62	0.70	0.40	0.26	0.52	0.44	0.26	0.26	0.23	0.26	0.19
RFE_DT	0.32	0.68	0.69	0.69	0.28	0.32	0.30	0.48	0.32	0.32	0.28	0.32	0.25
Boruta_DT	0.33	0.65	0.64	0.64	0.53	0.33	0.72	0.49	0.33	0.33	0.30	0.33	0.28
RFE_MLP	0.33	0.65	0.55	0.61	0.28	0.33	0.30	0.50	0.33	0.33	0.30	0.33	0.27
MRMR_DT	0.52	0.64	0.73	0.65	0.42	0.52	0.58	0.46	0.52	0.52	0.36	0.52	0.46
MRMR_SVM	0.35	0.63	0.73	0.67	0.32	0.35	0.43	0.30	0.35	0.35	0.30	0.35	0.38
Boruta_SVM	0.37	0.62	0.73	0.67	0.34	0.37	0.55	0.26	0.37	0.37	0.16	0.37	0.24
MRMR_MLP	0.24	0.55	0.49	0.55	0.50	0.24	0.71	0.32	0.24	0.24	0.18	0.24	0.19
RFE_SVM	0.28	0.52	0.50	0.52	0.47	0.28	0.66	0.38	0.28	0.28	0.24	0.28	0.25
Boruta_MLP	0.17	0.50	0.45	0.50	0.29	0.17	0.38	0.25	0.17	0.17	0.08	0.17	0.06

RFE: recursive feature elimination, MRMR: minimum redundancy maximum relevance, MLP: multi-layer perceptron, DT: decision tree, KNN: k-nearest neighbors, NB: naïve bayes, RF: random forest, SVM: support vector machine, XGB: extreme gradient boosting.

Table S14. Comparison of performance between all the models trained using robust features against segmentation batch extracted from Zoomed dataset and tested on Zoomed etxternal dataset.

		AUC			Precision			Recall			F1-score		
Model	Accuracy	macro	micro	weighted	macro	micro	weighted	macro	micro	weighted	macro	micro	weighted
RFE_SVM	0.73	0.86	0.91	0.88	0.62	0.73	0.75	0.63	0.73	0.73	0.62	0.73	0.74
RFE_XGB	0.61	0.86	0.87	0.87	0.60	0.61	0.73	0.64	0.61	0.61	0.56	0.61	0.62
RFE_RF	0.60	0.85	0.87	0.87	0.59	0.60	0.72	0.64	0.60	0.60	0.55	0.60	0.60
MRMR_RF	0.60	0.85	0.86	0.86	0.61	0.60	0.76	0.64	0.60	0.60	0.55	0.60	0.59
RFE_NB	0.66	0.84	0.89	0.87	0.59	0.66	0.71	0.60	0.66	0.66	0.57	0.66	0.66
MRMR_XGB	0.64	0.84	0.87	0.86	0.56	0.64	0.70	0.61	0.64	0.64	0.56	0.64	0.64
RFE_KNN	0.66	0.83	0.88	0.85	0.59	0.66	0.73	0.61	0.66	0.66	0.58	0.66	0.68
MRMR_NB	0.64	0.82	0.83	0.85	0.56	0.64	0.71	0.60	0.64	0.64	0.55	0.64	0.65
MRMR_KNN	0.51	0.81	0.82	0.81	0.49	0.51	0.60	0.58	0.51	0.51	0.48	0.51	0.52
Boruta_NB	0.50	0.80	0.81	0.82	0.59	0.50	0.74	0.59	0.50	0.50	0.49	0.50	0.54
Boruta_XGB	0.50	0.80	0.77	0.81	0.63	0.50	0.81	0.62	0.50	0.50	0.50	0.50	0.55
MRMR_DT	0.51	0.80	0.82	0.81	0.60	0.51	0.76	0.63	0.51	0.51	0.51	0.51	0.55
Boruta_RF	0.49	0.79	0.75	0.79	0.60	0.49	0.77	0.61	0.49	0.49	0.49	0.49	0.53
MRMR_SVM	0.52	0.79	0.80	0.81	0.44	0.52	0.58	0.41	0.52	0.52	0.37	0.52	0.51
Boruta_DT	0.50	0.78	0.80	0.79	0.60	0.50	0.75	0.59	0.50	0.50	0.49	0.50	0.52
Boruta_KNN	0.45	0.77	0.76	0.76	0.49	0.45	0.63	0.53	0.45	0.45	0.43	0.45	0.47
RFE_DT	0.49	0.76	0.80	0.75	0.54	0.49	0.65	0.58	0.49	0.49	0.49	0.49	0.53
Boruta_MLP	0.65	0.75	0.76	0.76	0.63	0.65	0.76	0.63	0.65	0.65	0.59	0.65	0.68
Boruta_SVM	0.36	0.75	0.75	0.76	0.34	0.36	0.54	0.25	0.36	0.36	0.14	0.36	0.20
MRMR_MLP	0.56	0.73	0.70	0.72	0.57	0.56	0.72	0.60	0.56	0.56	0.51	0.56	0.58
RFE_MLP	0.60	0.69	0.73	0.69	0.58	0.60	0.69	0.54	0.60	0.60	0.49	0.60	0.55

RFE: recursive feature elimination, MRMR: minimum redundancy maximum relevance, MLP: multi-layer perceptron, DT: decision tree, KNN: k-nearest neighbors, NB: naïve bayes, RF: random forest, SVM: support vector machine, XGB: extreme gradient boosting.

Table S15. Comparison of performance between all the models trained using robust features against segmentation batch extracted from Baseline dataset and tested on Baseline etxternal dataset.

		AUC			Precision			Recall			F1-score		
Model	Accuracy	macro	micro	weighted	macro	micro	weighted	macro	micro	weighted	macro	micro	weighted
RFE_RF	0.58	0.86	0.82	0.85	0.66	0.58	0.71	0.64	0.58	0.58	0.59	0.58	0.57
RFE_NB	0.62	0.85	0.85	0.85	0.63	0.62	0.65	0.65	0.62	0.62	0.62	0.62	0.62
Boruta_XGB	0.54	0.84	0.80	0.84	0.62	0.54	0.64	0.61	0.54	0.54	0.55	0.54	0.53
RFE_SVM	0.63	0.84	0.83	0.83	0.67	0.63	0.70	0.68	0.63	0.63	0.64	0.63	0.63
RFE_XGB	0.60	0.83	0.81	0.82	0.65	0.60	0.69	0.66	0.60	0.60	0.60	0.60	0.58
MRMR_XGB	0.56	0.83	0.79	0.82	0.61	0.56	0.63	0.60	0.56	0.56	0.57	0.56	0.55
RFE_KNN	0.59	0.82	0.80	0.81	0.63	0.59	0.66	0.63	0.59	0.59	0.60	0.59	0.59
Boruta_DT	0.53	0.80	0.77	0.78	0.60	0.53	0.61	0.60	0.53	0.53	0.55	0.53	0.52
Boruta_RF	0.54	0.80	0.77	0.77	0.58	0.54	0.58	0.60	0.54	0.54	0.56	0.54	0.53
MRMR_NB	0.50	0.79	0.76	0.79	0.56	0.50	0.56	0.52	0.50	0.50	0.52	0.50	0.50
MRMR_RF	0.51	0.78	0.75	0.76	0.55	0.51	0.56	0.56	0.51	0.51	0.52	0.51	0.50
RFE_DT	0.53	0.77	0.74	0.76	0.59	0.53	0.61	0.59	0.53	0.53	0.55	0.53	0.53
RFE_MLP	0.61	0.75	0.74	0.75	0.66	0.61	0.69	0.63	0.61	0.61	0.62	0.61	0.63
Boruta_NB	0.48	0.74	0.73	0.75	0.48	0.48	0.49	0.48	0.48	0.48	0.46	0.48	0.47
Boruta_KNN	0.53	0.74	0.74	0.72	0.6	0.53	0.61	0.57	0.53	0.53	0.55	0.53	0.53
MRMR_KNN	0.48	0.72	0.73	0.70	0.57	0.48	0.58	0.52	0.48	0.48	0.51	0.48	0.49
MRMR_MLP	0.53	0.69	0.68	0.68	0.57	0.53	0.59	0.55	0.53	0.53	0.54	0.53	0.54
MRMR_SVM	0.47	0.69	0.67	0.67	0.56	0.47	0.57	0.52	0.47	0.47	0.50	0.47	0.48
MRMR_DT	0.40	0.69	0.69	0.67	0.34	0.40	0.33	0.41	0.40	0.40	0.32	0.40	0.30
Boruta_SVM	0.20	0.68	0.53	0.67	0.34	0.20	0.43	0.27	0.20	0.20	0.15	0.20	0.14
Boruta_MLP	0.36	0.60	0.57	0.58	0.37	0.36	0.39	0.42	0.36	0.36	0.32	0.36	0.28

RFE: recursive feature elimination, MRMR: minimum redundancy maximum relevance, MLP: multi-layer perceptron, DT: decision tree, KNN: k-nearest neighbors, NB: naïve bayes, RF: random forest, SVM: support vector machine, XGB: extreme gradient boosting.

Table S16. Comparison of performance between all the models trained using robust features against segmentation batch extracted from Zoomed dataset and tested on Baseline etxternal dataset.

		AUC			Precision			Recall			F1-score		
Model	Accuracy	macro	micro	weighted	macro	micro	weighted	macro	micro	weighted	macro	micro	weighted
RFE_NB	0.37	0.76	0.66	0.75	0.41	0.37	0.35	0.40	0.37	0.37	0.34	0.37	0.30
MRMR_RF	0.38	0.73	0.62	0.71	0.57	0.38	0.59	0.45	0.38	0.38	0.37	0.38	0.33
RFE_XGB	0.37	0.73	0.65	0.71	0.47	0.37	0.44	0.45	0.37	0.37	0.37	0.37	0.32
Boruta_DT	0.39	0.73	0.65	0.71	0.47	0.39	0.44	0.45	0.39	0.39	0.40	0.39	0.36
RFE_RF	0.37	0.73	0.63	0.70	0.49	0.37	0.47	0.44	0.37	0.37	0.37	0.37	0.33
Boruta_XGB	0.41	0.73	0.67	0.70	0.52	0.41	0.51	0.47	0.41	0.41	0.42	0.41	0.39
MRMR_XGB	0.39	0.73	0.65	0.71	0.66	0.39	0.71	0.46	0.39	0.39	0.38	0.39	0.34
Boruta_RF	0.34	0.72	0.63	0.69	0.46	0.34	0.44	0.40	0.34	0.34	0.35	0.34	0.31
MRMR_NB	0.35	0.71	0.64	0.70	0.41	0.35	0.40	0.39	0.35	0.35	0.35	0.35	0.32
RFE_DT	0.36	0.67	0.65	0.68	0.43	0.36	0.40	0.39	0.36	0.36	0.35	0.36	0.32
MRMR_DT	0.37	0.66	0.63	0.65	0.45	0.37	0.43	0.38	0.37	0.37	0.34	0.37	0.32
RFE_KNN	0.36	0.64	0.58	0.61	0.49	0.36	0.48	0.40	0.36	0.36	0.34	0.36	0.31
Boruta_KNN	0.22	0.60	0.58	0.58	0.38	0.22	0.37	0.27	0.22	0.22	0.25	0.22	0.22
MRMR_KNN	0.23	0.59	0.56	0.57	0.46	0.23	0.48	0.30	0.23	0.23	0.26	0.23	0.22
RFE_MLP	0.32	0.55	0.54	0.55	0.37	0.32	0.32	0.33	0.32	0.32	0.26	0.32	0.24
Boruta_SVM	0.16	0.54	0.44	0.52	0.04	0.16	0.02	0.25	0.16	0.16	0.07	0.16	0.04
MRMR_MLP	0.23	0.52	0.48	0.50	0.38	0.23	0.37	0.29	0.23	0.23	0.26	0.23	0.23
Boruta_MLP	0.21	0.50	0.47	0.49	0.41	0.21	0.39	0.27	0.21	0.21	0.23	0.21	0.19
Boruta_NB	0.19	0.50	0.46	0.49	0.19	0.19	0.19	0.20	0.19	0.19	0.19	0.19	0.18
RFE_SVM	0.20	0.49	0.47	0.46	0.31	0.20	0.27	0.27	0.20	0.20	0.19	0.20	0.15
MRMR_SVM	0.15	0.49	0.42	0.47	0.30	0.15	0.25	0.22	0.15	0.15	0.10	0.15	0.07

RFE: recursive feature elimination, MRMR: minimum redundancy maximum relevance, MLP: multi-layer perceptron, DT: decision tree, KNN: k-nearest neighbors, NB: naïve bayes, RF: random forest, SVM: support vector machine, XGB: extreme gradient boosting.

Table S17. Comparison of performance between all the models trained using mutually robust features extracted from Baseline dataset and tested on Zoomed etxternal dataset.

		AUC			Precision			Recall			F1-score		
Model	Accuracy	macro	micro	weighted	macro	micro	weighted	macro	micro	weighted	macro	micro	weighted
Boruta_XGB	0.37	0.80	0.73	0.81	0.52	0.37	0.71	0.52	0.37	0.37	0.34	0.37	0.35
MRMR_NB	0.47	0.80	0.78	0.83	0.62	0.47	0.80	0.61	0.47	0.47	0.48	0.47	0.52
MRMR_XGB	0.40	0.79	0.73	0.81	0.55	0.40	0.74	0.53	0.40	0.40	0.38	0.40	0.40
MRMR_KNN	0.43	0.77	0.73	0.78	0.54	0.43	0.70	0.55	0.43	0.43	0.42	0.43	0.45
Boruta_NB	0.48	0.76	0.75	0.78	0.53	0.48	0.69	0.58	0.48	0.48	0.46	0.48	0.49
Boruta_KNN	0.42	0.76	0.72	0.76	0.48	0.42	0.64	0.52	0.42	0.42	0.39	0.42	0.43
MRMR_RF	0.35	0.75	0.69	0.76	0.53	0.35	0.72	0.51	0.35	0.35	0.33	0.35	0.33
Boruta_RF	0.35	0.73	0.67	0.74	0.52	0.35	0.70	0.50	0.35	0.35	0.32	0.35	0.31
MRMR_SVM	0.37	0.73	0.75	0.76	0.40	0.37	0.58	0.37	0.37	0.37	0.30	0.37	0.39
RFE_XGB	0.31	0.73	0.62	0.74	0.51	0.31	0.71	0.45	0.31	0.31	0.27	0.31	0.26
RFE_RF	0.31	0.72	0.61	0.73	0.49	0.31	0.67	0.42	0.31	0.31	0.26	0.31	0.25
RFE_NB	0.27	0.70	0.60	0.72	0.48	0.27	0.67	0.40	0.27	0.27	0.23	0.27	0.22
RFE_KNN	0.32	0.69	0.60	0.71	0.51	0.32	0.73	0.38	0.32	0.32	0.27	0.32	0.31
Boruta_DT	0.32	0.68	0.69	0.68	0.28	0.32	0.30	0.48	0.32	0.32	0.28	0.32	0.25
Boruta_SVM	0.39	0.68	0.73	0.72	0.34	0.39	0.55	0.27	0.39	0.39	0.18	0.39	0.26
MRMR_DT	0.30	0.68	0.66	0.68	0.27	0.30	0.29	0.47	0.30	0.30	0.27	0.30	0.24
RFE_DT	0.31	0.66	0.66	0.68	0.27	0.31	0.30	0.45	0.31	0.31	0.27	0.31	0.25
Boruta_MLP	0.26	0.58	0.51	0.56	0.28	0.26	0.38	0.39	0.26	0.26	0.21	0.26	0.20
RFE_MLP	0.20	0.56	0.47	0.55	0.33	0.20	0.41	0.35	0.20	0.20	0.16	0.20	0.12
MRMR_MLP	0.18	0.52	0.45	0.51	0.18	0.18	0.24	0.28	0.18	0.18	0.11	0.18	0.08
RFE_SVM	0.30	0.52	0.54	0.54	0.52	0.30	0.74	0.39	0.30	0.30	0.26	0.30	0.27

RFE: recursive feature elimination, MRMR: minimum redundancy maximum relevance, MLP: multi-layer perceptron, DT: decision tree, KNN: k-nearest neighbors, NB: naïve bayes, RF: random forest, SVM: support vector machine, XGB: extreme gradient boosting.

Table S18. Comparison of performance between all the models trained using mutually robust features extracted from Zoomed dataset and tested on Baseline etxternal dataset.

		AUC			Precision			Recall			F1-score		
Model	Accuracy	macro	micro	weighted	macro	micro	weighted	macro	micro	weighted	macro	micro	weighted
Boruta_MLP	0.70	0.74	0.80	0.76	0.61	0.70	0.71	0.59	0.70	0.70	0.6	0.70	0.69
MRMR_NB	0.67	0.83	0.88	0.86	0.57	0.67	0.69	0.59	0.67	0.67	0.58	0.67	0.67
MRMR_MLP	0.65	0.73	0.76	0.77	0.62	0.65	0.75	0.59	0.65	0.65	0.58	0.65	0.69
Boruta_NB	0.63	0.82	0.87	0.84	0.57	0.63	0.66	0.58	0.63	0.63	0.57	0.63	0.64
MRMR_KNN	0.62	0.82	0.85	0.84	0.55	0.62	0.68	0.61	0.62	0.62	0.56	0.62	0.63
Boruta_KNN	0.61	0.80	0.83	0.81	0.54	0.61	0.68	0.58	0.61	0.61	0.53	0.61	0.62
RFE_DT	0.57	0.72	0.82	0.75	0.56	0.57	0.70	0.53	0.57	0.57	0.48	0.57	0.53
MRMR_XGB	0.56	0.84	0.84	0.86	0.58	0.56	0.74	0.61	0.56	0.56	0.52	0.56	0.59
RFE_NB	0.56	0.80	0.83	0.81	0.50	0.56	0.61	0.55	0.56	0.56	0.50	0.56	0.56
RFE_RF	0.55	0.81	0.79	0.82	0.59	0.55	0.68	0.55	0.55	0.55	0.49	0.55	0.49
RFE_KNN	0.54	0.77	0.79	0.76	0.49	0.54	0.59	0.52	0.54	0.54	0.46	0.54	0.50
RFE_MLP	0.53	0.76	0.69	0.74	0.68	0.53	0.84	0.66	0.53	0.53	0.55	0.53	0.60
RFE_XGB	0.53	0.81	0.81	0.84	0.53	0.53	0.65	0.57	0.53	0.53	0.48	0.53	0.50
Boruta_DT	0.52	0.79	0.81	0.81	0.60	0.52	0.76	0.60	0.52	0.52	0.50	0.52	0.54
MRMR_DT	0.51	0.81	0.80	0.83	0.64	0.51	0.81	0.63	0.51	0.51	0.51	0.51	0.55
MRMR_RF	0.51	0.83	0.81	0.84	0.60	0.51	0.76	0.63	0.51	0.51	0.49	0.51	0.51
Boruta_RF	0.50	0.82	0.81	0.83	0.57	0.50	0.72	0.60	0.50	0.50	0.48	0.50	0.52
MRMR_SVM	0.49	0.80	0.80	0.82	0.41	0.49	0.56	0.36	0.49	0.49	0.32	0.49	0.45
Boruta_XGB	0.45	0.83	0.80	0.85	0.57	0.45	0.73	0.59	0.45	0.45	0.44	0.45	0.46
RFE_SVM	0.37	0.72	0.68	0.73	0.46	0.37	0.63	0.26	0.37	0.37	0.16	0.37	0.23
Boruta_SVM	0.36	0.79	0.72	0.81	0.34	0.36	0.54	0.25	0.36	0.36	0.14	0.36	0.20

RFE: recursive feature elimination, MRMR: minimum redundancy maximum relevance, MLP: multi-layer perceptron, DT: decision tree, KNN: k-nearest neighbors, NB: naïve bayes, RF: random forest, SVM: support vector machine, XGB: extreme gradient boosting.

Table S19. Comparison of performance between all the models trained using mutually robust features extracted from Baseline dataset and tested on Baseline etxternal dataset.

		AUC			Precision			Recall			F1-score		
Model	Accuracy	macro	micro	weighted	macro	micro	weighted	macro	micro	weighted	macro	micro	weighted
RFE_MLP	0.69	0.80	0.79	0.79	0.69	0.69	0.71	0.71	0.69	0.69	0.69	0.69	0.69
RFE_SVM	0.64	0.81	0.80	0.80	0.66	0.64	0.69	0.68	0.64	0.64	0.64	0.64	0.63
RFE_NB	0.62	0.86	0.84	0.87	0.65	0.62	0.67	0.64	0.62	0.62	0.62	0.62	0.61
RFE_XGB	0.60	0.85	0.82	0.84	0.65	0.60	0.68	0.66	0.60	0.60	0.61	0.60	0.58
RFE_KNN	0.60	0.82	0.81	0.81	0.64	0.60	0.67	0.65	0.60	0.60	0.61	0.60	0.59
Boruta_MLP	0.57	0.71	0.71	0.71	0.74	0.57	0.74	0.57	0.57	0.57	0.50	0.57	0.52
MRMR_XGB	0.57	0.82	0.78	0.81	0.63	0.57	0.64	0.63	0.57	0.57	0.58	0.57	0.56
MRMR_MLP	0.57	0.72	0.71	0.71	0.62	0.57	0.64	0.58	0.57	0.57	0.53	0.57	0.54
RFE_RF	0.56	0.85	0.81	0.84	0.63	0.56	0.66	0.62	0.56	0.56	0.58	0.56	0.56
Boruta_RF	0.56	0.83	0.79	0.82	0.61	0.56	0.62	0.62	0.56	0.56	0.57	0.56	0.54
MRMR_RF	0.56	0.83	0.78	0.81	0.61	0.56	0.63	0.62	0.56	0.56	0.57	0.56	0.55
Boruta_DT	0.55	0.78	0.76	0.77	0.61	0.55	0.63	0.61	0.55	0.55	0.57	0.55	0.55
Boruta_XGB	0.54	0.84	0.80	0.83	0.60	0.54	0.62	0.61	0.54	0.54	0.55	0.54	0.53
RFE_DT	0.54	0.78	0.76	0.77	0.61	0.54	0.63	0.61	0.54	0.54	0.56	0.54	0.53
MRMR_DT	0.52	0.77	0.74	0.75	0.57	0.52	0.58	0.57	0.52	0.52	0.53	0.52	0.51
Boruta_KNN	0.50	0.77	0.75	0.75	0.57	0.50	0.58	0.53	0.50	0.50	0.51	0.50	0.49
MRMR_KNN	0.51	0.76	0.74	0.75	0.58	0.51	0.58	0.55	0.51	0.51	0.52	0.51	0.49
MRMR_SVM	0.47	0.76	0.70	0.74	0.55	0.47	0.56	0.54	0.47	0.47	0.47	0.47	0.42
MRMR_NB	0.40	0.79	0.72	0.78	0.50	0.40	0.48	0.42	0.40	0.40	0.40	0.40	0.38
Boruta_NB	0.38	0.79	0.72	0.80	0.48	0.38	0.48	0.39	0.38	0.38	0.35	0.38	0.33
Boruta_SVM	0.29	0.78	0.62	0.77	0.36	0.29	0.43	0.35	0.29	0.29	0.22	0.29	0.21

RFE: recursive feature elimination, MRMR: minimum redundancy maximum relevance, MLP: multi-layer perceptron, DT: decision tree, KNN: k-nearest neighbors, NB: naïve bayes, RF: random forest, SVM: support vector machine, XGB: extreme gradient boosting.

Table S20. Comparison of performance between all the models trained using mutually robust features extracted from Zoomed dataset and tested on Baseline etxternal dataset.

		AUC			Precision			Recall			F1-score		
Model	Accuracy	macro	micro	weighted	macro	micro	weighted	macro	micro	weighted	macro	micro	weighted
MRMR_RF	0.69	0.76	0.67	0.73	0.49	0.39	0.46	0.46	0.69	0.39	0.40	0.39	0.36
Boruta_XGB	0.64	0.75	0.69	0.73	0.51	0.43	0.49	0.49	0.64	0.43	0.44	0.43	0.40
Boruta_RF	0.62	0.75	0.66	0.72	0.47	0.38	0.44	0.44	0.62	0.38	0.39	0.38	0.35
MRMR_XGB	0.60	0.74	0.68	0.72	0.53	0.44	0.52	0.50	0.60	0.44	0.44	0.44	0.40
RFE_XGB	0.60	0.74	0.67	0.72	0.53	0.38	0.53	0.45	0.60	0.38	0.38	0.38	0.33
RFE_NB	0.57	0.73	0.68	0.73	0.42	0.38	0.40	0.40	0.57	0.38	0.35	0.38	0.32
RFE_RF	0.57	0.72	0.64	0.70	0.51	0.34	0.52	0.41	0.57	0.34	0.34	0.34	0.30
MRMR_DT	0.57	0.72	0.65	0.70	0.51	0.42	0.49	0.47	0.57	0.42	0.44	0.42	0.40
Boruta_DT	0.56	0.72	0.65	0.70	0.45	0.38	0.41	0.44	0.56	0.38	0.39	0.38	0.34
MRMR_NB	0.56	0.70	0.61	0.71	0.41	0.29	0.4	0.31	0.56	0.29	0.22	0.29	0.21
RFE_KNN	0.56	0.70	0.63	0.69	0.57	0.41	0.60	0.45	0.56	0.41	0.38	0.41	0.35
RFE_DT	0.55	0.69	0.62	0.68	0.40	0.37	0.35	0.44	0.55	0.37	0.36	0.37	0.31
MRMR_KNN	0.54	0.67	0.63	0.66	0.44	0.38	0.42	0.41	0.54	0.38	0.35	0.38	0.32
Boruta_NB	0.54	0.67	0.57	0.67	0.35	0.23	0.32	0.23	0.54	0.23	0.14	0.23	0.14
MRMR_SVM	0.52	0.64	0.52	0.61	0.43	0.21	0.42	0.29	0.52	0.21	0.16	0.21	0.14
Boruta_KNN	0.50	0.6	0.59	0.57	0.43	0.35	0.39	0.38	0.50	0.35	0.33	0.35	0.30
Boruta_SVM	0.51	0.58	0.47	0.57	0.06	0.17	0.05	0.25	0.51	0.17	0.08	0.17	0.05
RFE_SVM	0.47	0.58	0.46	0.53	0.07	0.17	0.05	0.24	0.47	0.17	0.09	0.17	0.07
RFE_MLP	0.40	0.57	0.55	0.55	0.46	0.33	0.43	0.37	0.40	0.33	0.32	0.33	0.29
MRMR_MLP	0.38	0.56	0.55	0.55	0.43	0.32	0.39	0.36	0.38	0.32	0.27	0.32	0.25
Boruta_MLP	0.29	0.50	0.48	0.50	0.60	0.23	0.66	0.26	0.29	0.23	0.20	0.23	0.18

RFE: recursive feature elimination, MRMR: minimum redundancy maximum relevance, MLP: multi-layer perceptron, DT: decision tree, KNN: k-nearest neighbors, NB: naïve bayes, RF: random forest, SVM: support vector machine, XGB: extreme gradient boosting.
